# Placental endocrine function shapes cerebellar development and social behavior

**DOI:** 10.1038/s41593-021-00896-4

**Published:** 2021-08-16

**Authors:** Claire-Marie Vacher, Helene Lacaille, Jiaqi J. O’Reilly, Jacquelyn Salzbank, Dana Bakalar, Sonia Sebaoui, Philippe Liere, Cheryl Clarkson-Paredes, Toru Sasaki, Aaron Sathyanesan, Panagiotis Kratimenos, Jacob Ellegood, Jason P. Lerch, Yuka Imamura, Anastas Popratiloff, Kazue Hashimoto-Torii, Vittorio Gallo, Michael Schumacher, Anna A. Penn

**Affiliations:** 1 Department of Pediatrics, Columbia University, New York-Presbyterian Morgan Stanley Children’s Hospital, New York, NY USA; 2 National Institutes of Health, Bethesda, MD USA; 3 Center for Neuroscience Research, Children’s National Health System, Washington, DC USA; 4 U1195 INSERM, Paris-Saclay University, Le Kremlin‐Bicêtre Cedex, France; 5 The George Washington University, Nanofabrication and Imaging Center, Washington, DC USA; 6 The George Washington University, SMHS, Anatomy & Cell Biology, Washington, DC USA; 7 Mouse Imaging Centre (MICe), Hospital for Sick Children, Toronto, ON Canada; 8 Wellcome Centre for Integrative Neuroimaging (WIN), Nuffield Department of Clinical Neurosciences, University of Oxford, John Radcliffe Hospital, Oxford, UK; 9 Department of Biochemistry and Molecular Biology, Pennsylvania State University College of Medicine, Pittsburgh, PA USA; 10 The George Washington University School of Medicine and Health Sciences, Pediatrics, Washington, DC USA

**Keywords:** Development of the nervous system, Diseases of the nervous system

## Abstract

Compromised placental function or premature loss has been linked to diverse neurodevelopmental disorders. Here we show that placenta allopregnanolone (ALLO), a progesterone-derived GABA-A receptor (GABA_A_R) modulator, reduction alters neurodevelopment in a sex-linked manner. A new conditional mouse model, in which the gene encoding ALLO’s synthetic enzyme (*akr1c14*) is specifically deleted in trophoblasts, directly demonstrated that placental ALLO insufficiency led to cerebellar white matter abnormalities that correlated with autistic-like behavior only in male offspring. A single injection of ALLO or muscimol, a GABA_A_R agonist, during late gestation abolished these alterations. Comparison of male and female human preterm infant cerebellum also showed sex-linked myelination marker alteration, suggesting similarities between mouse placental ALLO insufficiency and human preterm brain development. This study reveals a new role for a placental hormone in shaping brain regions and behaviors in a sex-linked manner. Placental hormone replacement might offer novel therapeutic opportunities to prevent later neurobehavioral disorders.

## Abstract (web summary)

Placental dysfunction has been implicated in abnormal neurodevelopment. Vacher et al. found that loss of a neuroactive hormone from the placenta alters brain development in a regional and sex-linked manner, resulting in autism-like behaviors in male offspring.

## Main

Neuroplacentology is a term coined to describe an emerging research area that aims to understand the influence of placental function on the developing brain. Placental dysfunction or pathology has been associated with abnormal neurodevelopment^1^, but the causal mechanisms remain largely unknown. Much research connecting placental compromise to fetal brain injury has focused on gas exchange or nutritional programming, neglecting the placenta’s essential neuroendocrine role. Many events, including infection, malnutrition and genetic abnormalities, can disrupt placental function or, as in preterm birth, can abruptly change the hormonal environment of the developing brain. Such changes might alter early brain development or increase the susceptibility of the immature brain to damage.

ALLO, a major GABAergic neurosteroid, might be one key placental hormone in shaping fetal brain. ALLO is synthesized from progesterone and is a positive allosteric modulator of GABA_A_Rs that binds to specific receptor sites and prolongs the opening of the GABA-gated Cl- channel^2^. In the adult brain, ALLO enhances GABAergic inhibition, producing sedative, anxiolytic, anesthetic and anticonvulsant effects^3^. A large body of evidence suggests that ALLO, through GABA_A_R signaling, is also a potent regulator of many neurodevelopmental processes, including neurogenesis, neuritogenesis, cell survival, synapse stabilization and myelination^4^. In fetal sheep and guinea pigs, brain ALLO is increased in response to immune challenge, hypoxia and other acute prenatal stressors. Blocking this increase pharmacologically with finasteride, a 5α-reductase inhibitor that also blocks ALLO production, leads to increased apoptosis, excitotoxicity and impaired myelination, particularly in males^5^. These observations support an endogenous neuroprotective role for ALLO and a potential therapeutic efficacy of ALLO analogs as central nervous system drugs but do not directly connect specific placental endocrine functions to changes in neurodevelopment.

Our novel mouse model of specific placental ALLO reduction allowed direct investigation of the neurodevelopmental effect of altered placental endocrine function at a molecular, cellular and functional level. A combination of microscopy, gene and protein expression analysis, neurobehavioral assessments and pharmacological rescue in this model shows that placenta ALLO insufficiency leads to cerebellar myelination abnormalities that correlate with autistic-like behaviors in a sex-linked manner. Understanding how specific placental hormones shape normal brain development and how placental loss or dysfunction contributes to the neurological impairments in those born extremely preterm or after compromised pregnancies lays the groundwork for developing hormone replacement strategies to maintain the normal developmental milieu and protect the brain from further injury.

## Results

### *Akr1c14* deletion in *Cyp19*^*+*^ cells impairs placental ALLO production

**Fig. 1:**
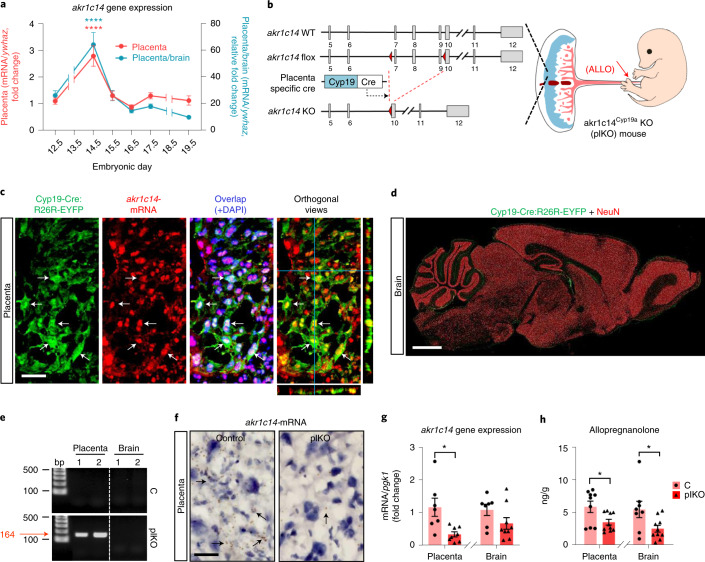
Conditional deletion of *akr1c14* in Cyp19a-expressing trophoblasts results in reduced ALLO levels in the fetal brain. **a**, qRT–PCR for *akr1c14* in wild-type (WT) mice. Data presented as mean fold changes ± s.e.m. E12.5: *n* = 12 C and 12 plKO; E14.5: *n* = 12 C and 12 plKO; E15.5: *n* = 10 C and 8 plKO; E16.5: *n* = 7 C and 7 plKO; E17.5: *n* = 12 C and 12 plKO; E19.5: *n* = 12 C and 11 plKO. One-way ANOVA with Dunnett’s multiple comparisons (*P* < 0.0001 compared to E12.5 value). There was no significant difference between males and females. *ywhaz*, tyrosine 3-monooxygenase/tryptophan 5-monooxygenase activation protein zeta. **b**, *akr1c14* genetic locus before and after recombination. Exons 7–9 are conditionally targeted. Cyp19a promoter drives expression of Cre in the placenta only. LoxP sites are indicated by red triangles and exons 5–12 by gray boxes. **c**, In situ hybridization for a*kr1c14* in placenta from Cyp19a-Cre:R26R-EYFP mice at E17.5. Arrows show co-localizations. Scale bar, 50 µm. **d**, Sagittal section of a Cyp19a-Cre:R26R-EYFP mouse brain immunostained against YFP (green) and NeuN (red). No Cyp19a promotor activity was evidenced in the brain at P30. Scale bar, 1 mm. **e**, A 164-bp-long PCR product ascertains the presence of the recombined LoxP site in the placenta of plKO mice. **f**, In situ hybridization for *akr1c14* in the placenta at E17.5. *akr1c14*-mRNA levels are drastically decreased in the spongiotrophoblasts (arrows) of plKO compared to C mice. Scale bar, 30 µm. **g**, qRT–PCR for *akr1c14* normalized to *pgk1* at E17.5. Data are presented as mean ± s.e.m. Two-tailed unpaired *t*-test with Welch’s correction (*P < 0.05). Placenta: *n* = 7 C and 8 plKO (*P* = 0.0247); Brain: *n* = 8 C and 9 plKO (*P* = 0.122). *Pgk1*, phosphoglycerate kinase 1. **h**, Mass spectrometry ALLO assays at E17.5. Data are presented as mean ± s.e.m. Two-tailed unpaired *t*-test with Welch’s correction (*P < 0.05). Placenta: *n* = 9 C and 10 plKO (*P* = 0.0315); Brain: *n* = 8 C and 10 plKO (*P* = 0.0329).

**Extended Data Fig. 1:**
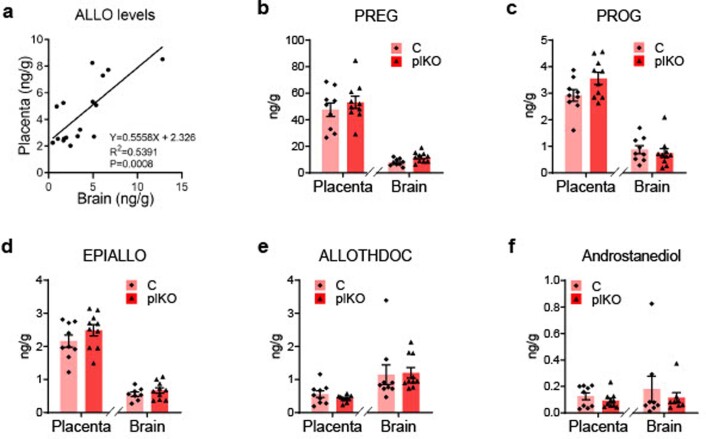
Chemical validation of the plKO model by mass spectrometry. **a**, Linear regression of ALLO levels in placenta and brain, all sexes and genotypes included. ALLO levels in placenta and brain show a positive correlation (p = 0.0008; n = 18). **b-f**, Mass spectrometry data in placenta and brain at E17.5. Data is presented as means ± SEM (n = 9 C and 10 plKO). Two-way ANOVA with Sidak’s multiple comparison test. Sex mixed as no sex effect was evidenced. ALLO, allopregnanolone; EPIALLO, epiallopregnanolone; PREG, pregnenolone; PROG, progesterone; ALLOTHDOC, allotetrahydrodeoxycorticosterone.

During fetal development, *akr1c14*, the gene encoding the ALLO-synthesizing enzyme 3α-hydroxysteroid dehydrogenase (3α-HSD), is primarily expressed in the placenta, with up to 60 times higher expression than in mouse brain, and peaks in the second half of gestation (Fig. 1a). To assess the neurodevelopmental role of placental ALLO, we generated *akr1c14*-floxed mice and crossed them with placenta-specific Cyp19a-Cre mice^6^ (Fig. 1b). Recombinase activity was confirmed specifically in placenta (Fig. 1c) but not in brain (Fig. 1d), as evidenced by crossing Cyp19-Cre with R26R-EYFP reporter mice. *Akr1c14*-mRNA co-localized with Cyp19a-Cre:R26R-EYFP-positive cells (arrows, Fig. 1c). Polymerase chain reaction (PCR) confirmed recombination of LoxP sites in placenta, but not brain, of akr1c14^Cyp19a^ knockout (KO) mice (plKO for placental conditional KO) and no recombination in placenta or brain of control (C) animals (Fig. 1e). In the plKO mice, *akr1c14* transcript was significantly reduced in the placenta but not brain (Fig. 1f,g). Direct steroid measurements revealed that ALLO was significantly reduced in the placenta and fetal brain in late gestation (Fig. 1h and Supplementary Table 1). The highest gestational conversion rate of ALLO (based on progesterone (PROG)/ALLO ratio) in C mice coincided with the *akr1c14* gene expression peak at embryonic day 14.5 (E14.5) (Supplementary Table 1 and Fig. 1a). Thus, *akr1c14* placental gene removal results in greatest placental ALLO insufficiency in the second half of mouse gestation (E14.5). The positive placenta–brain correlation of ALLO levels is consistent with placental provision of ALLO to the fetal brain (Extended Data Fig. 1a). Placental ALLO levels were not altered by sex (Extended Data Fig. 1a). ALLO precursors and other neuroactive steroids in the synthesis pathway were not altered (Supplementary Table 1 and Extended Data Fig. 1b–f), with the exception of a transient E14.5 reduction of placental allotetrahydrodeoxycorticosterone (ALLOTHDOC), which is also converted by placental 3α-HSD (Supplementary Table 1). Placental ALLOTHDOC concentrations are negligible when compared to ALLO levels, suggesting that major effects in the plKO are primarily due to placental ALLO insufficiency (Supplementary Table 1). Additionally, the equivalent and specific decrease in both brain and placenta of plKO ALLO levels supports the specificity of this model (Fig. 1h and Extended Data Fig. 1b–f).

### Placental ALLO loss alters postnatal cerebellar myelination

**Extended Data Fig. 2:**
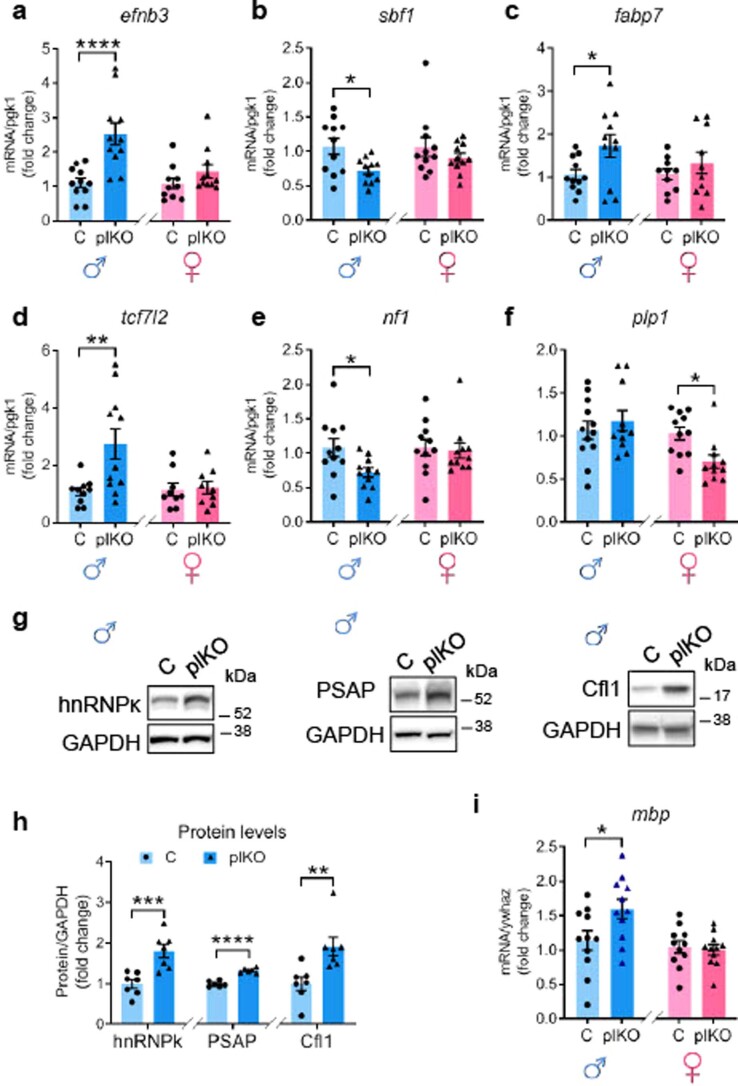
Molecular validation of the RNA sequencing cutoffs. Changes in mRNA (a-f) or protein (g-h) contents of selected cerebellar dysregulated genes after normalization. **a**, *efnb3*, ephrinB3 (RNAseq rank #26/1192 in males; up-regulated; q = 0.018; p = 3.41E-05). **b**, *sbf1*, SET Binding Factor 1 (RNAseq rank #82/1192 in males; down-regulated; q = 0.067; p = 0.00039). **c**, *fabp7*, Fatty Acid Binding Protein 7 (RNAseq rank #84/1192 in males; up-regulated; q = 0.0725; p = 0.00043). **d**, *tcf7l2*, transcription factor 7 like 2 (RNAseq rank #795/1192; up-regulated; q = 0.514; p = 0.029). **e**, *nf1*, Neurofibromin 1 (RNAseq rank #1189/1192 in males; down-regulated; q = 0.59; p = 0.0499). **f**, *plp1*, Proteolipid Protein 1 (RNAseq rank #64/389 in females; down-regulated; q = 1; p = 0.0054). Normalized qRT-PCR data (to *pgk1*) is presented as mean fold changes ± SEM, n = 10 samples/group.). Two-way ANOVA with Sidak’s multiple comparison test (a-f). *p < 0.05, **p < 0.01, ****p < 0.0001. **g-h**, Cerebellum protein contents determined by Western blot in males, and normalized to GAPDH levels. hnRNP_Κ_/Cfl1:n = 7 C and 7plKO; PSAP: n = 6 C and 6 plKO. Multiple unpaired t tests with Holm-Sidak multiple comparison test (**p < 0.01, ***p < 0.005, ****p < 0.0001). PSAP, prosaposin (RNAseq rank #950/1192 in males; up-regulated; q = 0.5329; p = 0.0359). Cfl1, cofilin-1 (RNAseq rank #27/1192 in males; up-regulated; q = 0.0192; p = 4.03E-05). hnRNPκ, heterogeneous nuclear ribonucleoprotein κ (rank #787; up-regulated; q = 0.509; p = 0.0284). **i**, *mbp*, myelin basic protein. Normalized qRT-PCR data (to *ywhaz*, Tyrosine 3-Monooxygenase/Tryptophan 5-Monooxygenase Activation Protein Zeta). Two-way ANOVA with Sidak’s multiple comparison test (n = 11 C and 11 plKO; *p < 0.05). See Extended Data Table 1 sheets 1 and 2 for list of ranked DEGs. Source data

**Extended Data Fig. 3:**
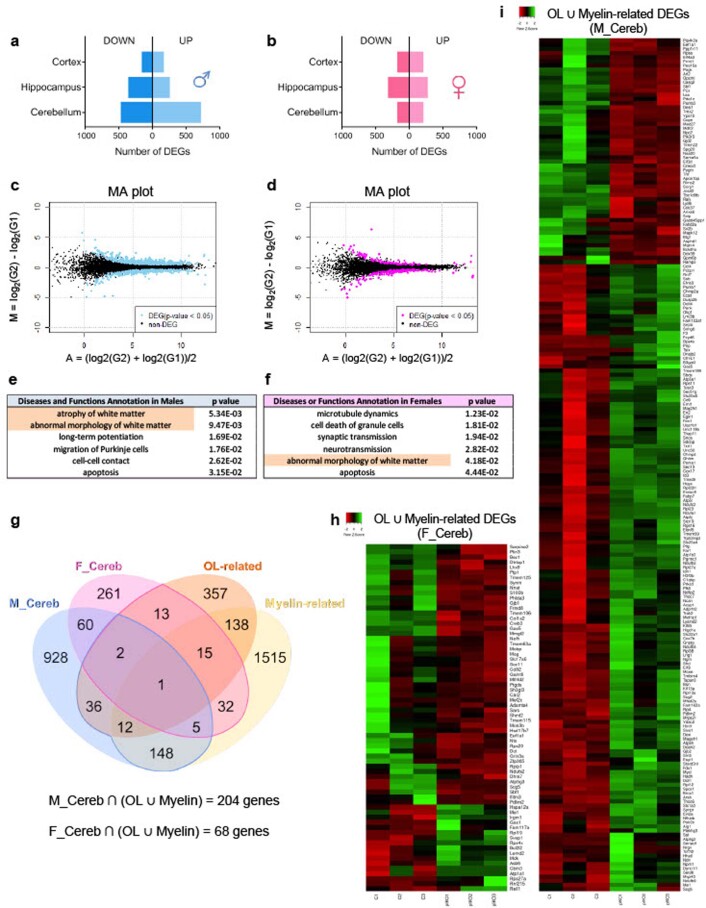
Long-term impact of placental ALLO insufficiency on brain transcriptome: highlight on the myelin-associated genes in the cerebellum. **a-b**, Number of differentially expressed genes (DEGs) that are up- or down-regulated in the cerebral cortex, hippocampus and cerebellum of plKO vs C mice at P30 in males (blue) and females (pink). The majority of DEGs were found in the male cerebellum. **c-d**, MA plots for the cerebellar RNAseq analysis of plKO vs C mice at P30. M, log ratio; A, mean average. DEGs are represented as colored dots (blue for males and pink for females). **e-f**, Top significant diseases and functions IPA annotations. **g**, Venn diagrams showing the overlaps between cerebellar DEGs in the plKO males (M_Cereb) and females (F_Cereb), and oligodendrocyte (OL) and myelin transcriptomes^3,4^. **h-i**, Heatmaps of OL-and myelin-related cerebellar DEGs in C and plKO samples. Genes and samples were hierarchically clustered based on Pearson distance of z-score data and average linkage. n = 3 independent samples/group^7,8^.

Unbiased RNA sequencing (RNA-seq) analysis of cerebral cortex, hippocampus and cerebellum of C and plKO mice at postnatal day 30 (P30) in both sexes was performed to define the most prominent postnatal molecular alterations associated with placental ALLO insufficiency. Threshold criteria used to identify significant differentially expressed genes (DEGs) were validated by RT–PCR and western blots (Extended Data Fig. 2). Male cerebellum was the brain structure affected most by placental ALLO deficiency as measured by the number of DEGs, which was 3–5 times higher than in any other brain region (Extended Data Fig. 3a,b and Supplementary Table 2, sheets 1 and 2). Cerebellar DEGs were equally distributed in up-regulated and down-regulated gene categories (Extended Data Fig. 3a–d). Ingenuity Pathway Analysis (IPA, Qiagen) identified white matter (WM)-associated pathways in the top categories of altered genes in both sexes (Extended Data Fig. 3e,f). Comparison of cerebellar DEGs to previous published oligodendrocyte (OL) and myelin transcriptomes^7,8^ revealed an overlap of 204 genes and 68 genes in males and females, respectively (Extended Data Fig. 3g). Striking sexual dimorphism was seen: male and female DEGs were qualitatively different, and most OL-/myelin-related DEGs were up-regulated in plKO males but down-regulated in plKO females (Extended Data Fig. 3h,i).

**Fig. 2:**
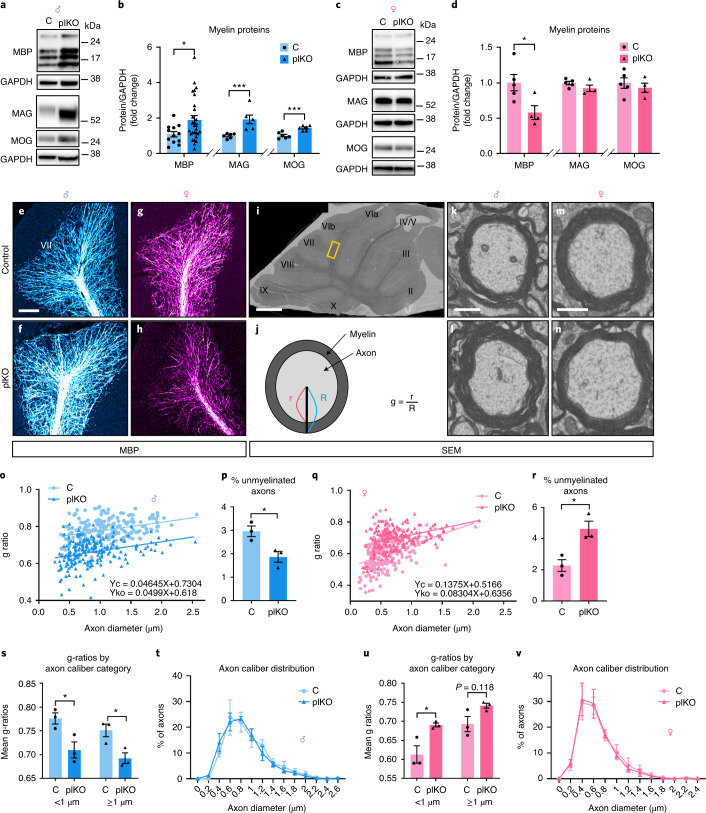
Placental ALLO insufficiency results in sex-linked cerebellar WM abnormalities at P30. **a**,**b**, Western blot analysis of myelin-related proteins in the male cerebellum. Data are presented as mean fold changes ± s.e.m. Multiple unpaired *t*-tests with Holm–Sidak multiple comparison test (*P < 0.05; ***P < 0.005). MBP: *n* = 12 C and 28 plKO (*P* = 0.038); MAG: *n* = 6 C and 6 plKO (*P* = 0.004); MOG: *n* = 6 C and 6plKO (*P* < 0.0001). **c**,**d**, Western blot analysis of myelin-related proteins in the female cerebellum. Data are presented as mean fold changes ± s.e.m. Multiple unpaired *t*-tests with Holm–Sidak multiple comparison test (*P < 0.05). MBP: *n* = 5 C and 4 plKO (*P* = 0.031); MAG: *n* = 5 C and 4 plKO (*P* = 0.18); MOG: *n* = 5 C and 4 plKO (*P* = 0.54). GAPDH, glyceraldehyde 3-phosphate dehydrogenase. **e**–**h**, Immunofluorescent staining of MBP in cerebellar lobule VII in males (**e** and **f**) and females (**g** and **h**). Scale bar, 150 μm. **i**, Scanning electron microscopy acquisition of a whole cerebellum ultrathin section showing the different cerebellar lobules (I–X) in a control mouse (×650 low magnification). The region of interest (inter-lobule VI–VII) for high-magnification acquisitions and g-ratio quantifications is indicated by the yellow rectangle. Scale bar, 300 μm. **j**, Schematic representation of an axon and its myelin sheath illustrating g-ratio. r, axon inner diameter; R, axon outer diameter. **k**–**n**, Representative scanning electron microscopy acquisitions (×20,000) of myelinated axons in inter-lobule VI–VII WM in males (**k** and **l**) and females (**m** and **n**). Scale bar, 300 nm. **o**, Scatter plot of g-ratios of >400 individual axons in males; data are from one representative control and one representative plKO mouse. Fitted lines are linear regressions. **p**, Percent of unmyelinated axons. Data are presented as mean ± s.e.m. Two-tailed unpaired Student’s *t*-test with Welch’s correction (*P < 0.05). *n* = 3 C and 3 plKO (*P* = 0.0275). **q**, Scatter plot of g-ratios of >400 individual axons in females; data are from one representative control and one representative plKO mouse. Fitted lines are linear regressions. **r**, Percent of unmyelinated axons. Data are presented as mean ± s.e.m. Two-tailed unpaired Student’s *t*-test with Welch’s correction (*P < 0.05). *n* = 3 C and 3 plKO (*P* = 0.0194). **s**, g-ratios by axon caliber category in males. Data are presented as mean ± s.e.m. Two-way ANOVA with Sidak’s multiple comparisons test (*P < 0.05). *n* = 3 C and 3 plKO (<1 µm: *P* = 0.0299; >1 µm: *P* = 0.0165). **t**, Axon caliber distribution in males (*n* = 3 C and 3 plKO). **u**, g-ratios by axon caliber category in females. Data are presented as mean ± s.e.m. Two-way ANOVA with Sidak’s multiple comparisons test (*P < 0.05). *n* = 3 C and 3 plKO <1 µm: *P* = 0.0162; >1 µm: *P* = 0.1185). **v**, Axon caliber distribution in females (*n* = 3 C and 3 plKO). Source data

Investigation of the cerebellar myelin proteins at P30 revealed major sex-linked changes: in plKO males, myelin basic protein (MBP), myelin-associated glycoprotein (MAG) and myelin oligodendrocyte glycoprotein (MOG) levels were all significantly increased (Fig. 2a,b), whereas, in plKO females, MBP cerebellar content was reduced (Fig. 2c,d)—changes confirmed by immunohistochemistry (Fig. 2e–h) and RT–PCR (Extended Data Fig. 2h). Similar sex-dependent MBP changes were previously described in the cerebellum of guinea pigs delivered preterm^9^. In addition, a migration downshift of MAG on western blots was observed in plKO males but not females (Fig. 2a,c), suggesting increased myelin maturation in males^10^. Further structural characterization of the cerebellar WM within lobule VI–VII by scanning electron microscopy (Fig. 2i) revealed thicker myelin (g-ratio measurements; Fig. 2j,k,l,o,s) and fewer unmyelinated axons in male plKOs (Fig. 2p). This increased myelin thickness was not associated with significant changes in axonal inner calibers (Fig. 2t). In contrast, cerebellar axons in plKO females had significantly thinner myelin sheath, and more axons were unmyelinated (Fig. 2m,n,q,r,u), although axonal inner diameters were unchanged (Fig. 2v) compared to C littermates.

**Extended Data Fig. 4:**
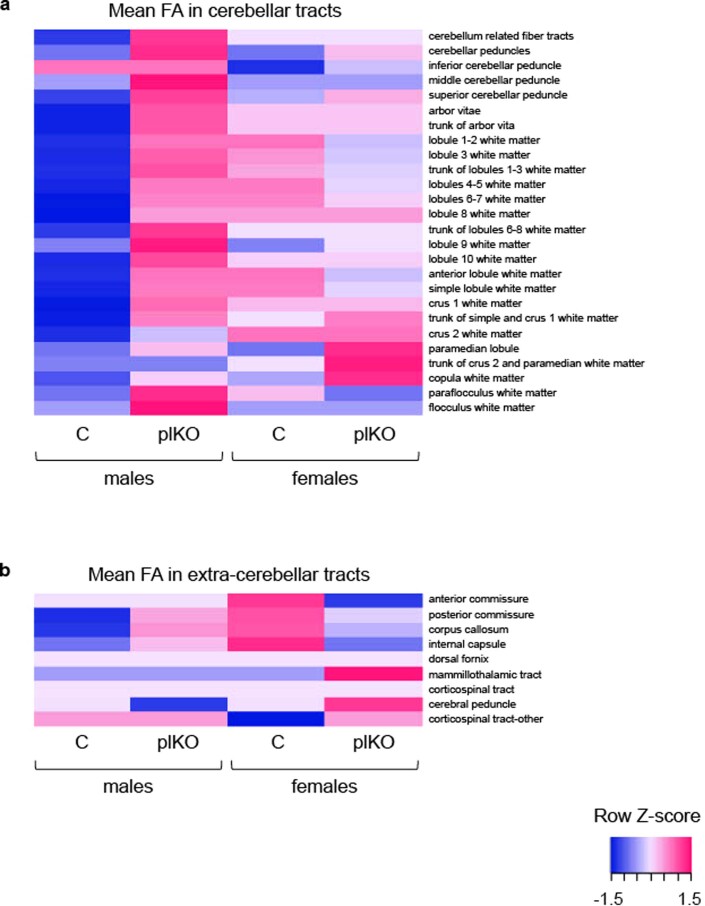
Diffusion Tensor Image (DTI) analysis at P30. **a**, Heatmap representing mean fractional anisotropies (FA) in the cerebellum related tracts. Mean FA is increased in the cerebellar WM of plKO males compared to C littermates. Data is presented in a hierarchical tree [region effect: F(25, 1014)=57.36, p < 0.0001; sex effect: F(1,1014)=2.097, p = 0.1479; genotype effect: F(1,1014)=16.99, p < 0.0001; sex × genotype interaction: F(1,1014)=17.92, p < 0.0001; 3-way ANOVA]. **b**, Heatmap representing mean FA in extra-cerebellar tracts. No significant change was evidenced between groups when compared for genotype or sex [region effect: F(8,351)=80.28, p < 0.0001; sex effect: F(1,351)=1.011, p = 0.3152; genotype effect: F(1,351)=0.1618, p = 0.6877; sex × genotype interaction: F(1,351)=2.589, p = 0.1021; 3-way ANOVA]. Males: n = 11 C and 13plKO; Females: n = 8 C and 11 plKO.

**Extended Data Fig. 5:**
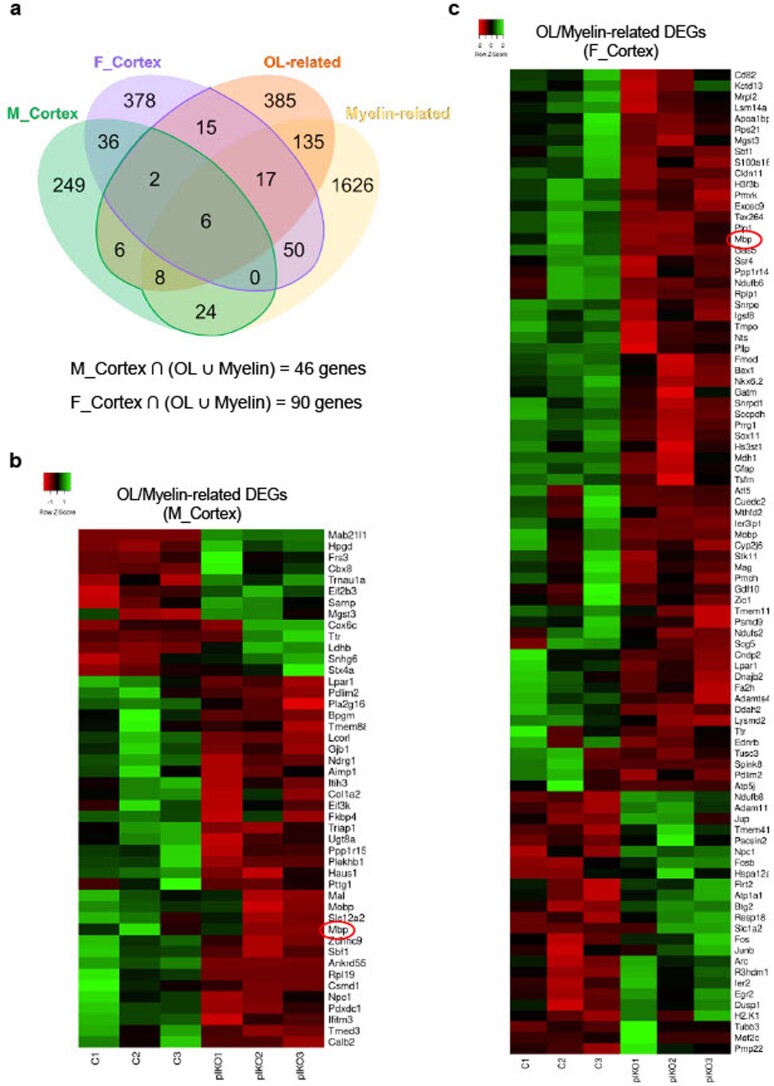
Long-term impact of placental ALLO insufficiency on cerebral cortex transcriptome at P30: highlight on the OL- and myelin-related genes. **a**, Venn diagrams showing the overlaps between cortical DEGs in the plKO males (M_Cortex) and females (F_Cortex), oligodendrocyte (OL) and myelin transcriptomes^3,4^. **b-c**, Heatmaps of OL-and myelin-related DEGs in the cerebral cortex of C and plKO samples. Genes and samples were hierarchically clustered based on Pearson distance of z-score data and average linkage. n = 3 independent samples/group.

A global comparison of regional, sex-linked and genotype-specific brain anatomy was performed by magnetic resonance imaging (MRI) at P30. Most notably, the mean fractional anisotropy (FA) of the cerebellar fiber tracts, measured by diffusion tensor imaging (DTI), was significantly increased in the plKO males, but not females, at P30 (Extended Data Fig. 4a; region effect: *F*_25,1014_ = 57.36, *P* < 0.0001; genotype effect: *F*_1,1014_ = 16.99, *P* < 0.0001; sex × genotype interaction: *F*_1,1014_ = 17.92, *P* < 0.0001; three-way analysis of variance (ANOVA)). In contrast, mean FAs in other WM tracts outside of the cerebellum were not significantly different in plKO mice compared to their C littermates (Extended Data Fig. 4b; region effect: *F*_8,351_ = 80.28, *P* < 0.0001; genotype effect: *F*_1,351_ = 0.1618, *P* = 0.6877; sex × genotype interaction: *F*_1,351_ = 2.589, *P* = 0.1021; three-way ANOVA). The male-specific increase in cerebellar FA is consistent with the enhanced degree of myelination^11^. OL-/myelin-related DEGs, including *mbp*, were mostly down-regulated in both male and female cerebral cortex in plKO mice (Extended Data Fig. 5). Both the DTI results and RNA-seq transcriptome patterns suggest overall sex-linked and regionally specific myelin abnormalities after placental ALLO loss.

### Postnatal OL differentiation rate diverges in plKO males and females

Cerebellar myelination begins in late mouse gestation and is primarily a postnatal process^12–14^, so placental endocrine disruption leading to myelination anomalies was unanticipated. The adjustment of myelin sheath thickness notably relies on the axon diameter, whose distribution was unchanged in plKO cerebellum (Fig. 2t,v) and the local density of myelin-competent OLs that are primarily produced before birth as OL progenitor cells (OPCs)^14^, at a time when ALLO is highest. ALLO is a potent positive allosteric modulator of GABA_A_R^2^. GABA_A_R signaling is associated with blockade of OPC proliferation^15,16^, so we tested the hypothesis that prenatal ALLO insufficiency enhances OLs production.

**Extended Data Fig. 6:**
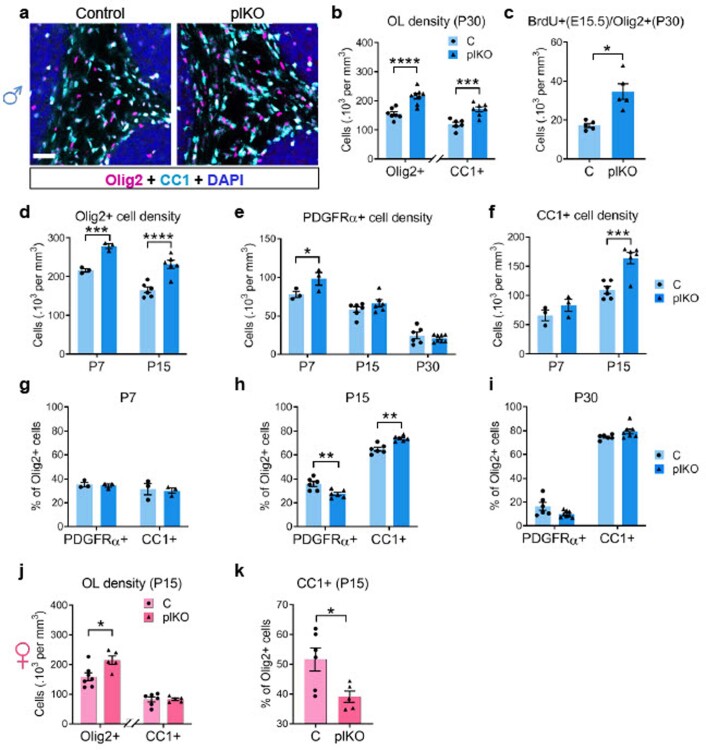
Oligodendrocyte lineage progression during the postnatal period. **a**, Immunofluorescent staining of Olig2 and CC1 in inter-lobule VI-VII WM at P30. Scale bar, 60 μm. **b**, Quantification of Olig2+ and CC1 + cell density in the male cerebellar WM at P30. Data presented as means ± SEM. Two-way ANOVA with Sidak’s multiple comparisons test (***p < 0.005, ****p < 0.0001). Olig2: n = 7 C and 8 plKO (p < 0.0001); CC1: n = 6 C and 7plKO (p = 0.0005). **c**, Quantification of BrdU + /Olig2+ cell density in the male cerebellar WM at P30. BrdU (50 mg/kg) was injected to dams at E15.5. Data presented as means ± SEM. Two-tailed unpaired Student’s t test with Welch’s correction (*p < 0.05). n = 5 C and 5plKO (p = 0.0107). **d**, Quantification of Olig2+ cell density in the male cerebellar WM at P7 and P15. Data is presented as means ± SEM. Two-way ANOVA with Sidak’s multiple comparisons test (***p < 0.005, ****p < 0.00010). P7: n = 3 mice/group (p = 0.0026); P15: n = 6 mice/group (p < 0.0001). The density of Olig2+ cells in the cerebellar WM is significantly increased in plKO males as compared with C littermates. **e**, OPC (PDGFRα + cell) density at P7, P15 and P30. Data is presented as means ± SEM. Two-way ANOVA with Sidak’s multiple comparisons test (*p < 0.05). P7: n = 3 mice/group (p = 0.0435); P15: n = 6 mice/group (p = 0.3644); P30: n = 6 C and 8 plKO (p = 0.8558). The density of OPCs in the cerebellar WM is transiently higher in plKO males as compared with C littermates at P7. **f**, Quantification of mature OL (CC1 + cell) density within the male cerebellar WM at P7 and P15. Data is presented as means ± SEM. Two-way ANOVA with Sidak’s multiple comparisons test (***p < 0.005). P7: n = 3 mice/group (p = 0.4951); P15: n = 6 mice/group (p < 0.0004). The density of mature OLs in the cerebellar WM is significantly increased in plKO males as compared with C littermates at P15. **g-i**, Ratios of OPCs (PDGFRα + cells) or mature OLs (CC1 + cells) within the total oligodendrocyte lineage (Olig2 + cells) in the male cerebellar WM across postnatal period. Data is presented as means ± SEM. Two-way ANOVA with Sidak’s multiple comparisons test (**p < 0.005). P7: n = 3 mice/group [p = 0.9741 (PDGFRα), p = 0.9393 (CC1)]; P15: n = 6 mice/group [p = 0.002 (PDGFRα), p = 0.001 (CC1)]; P30: n = 7 C and 8 plKO [p = 0.052 (PDGFRα), p = 0.17 (CC1)]. At P15, a transient acceleration of the OL maturation is observed in the male plKO. **j**, Quantification of total OL lineage (Olig2 + ) and mature OL (CC1 + ) cell densities in the female cerebellar WM at P15. Data is presented as means ± SEM. Two-way ANOVA with Sidak’s multiple comparisons test (*p < 0.01). Olig2 + : n = 7 C and 5 plKO (p = 0.0058); CC1 + : n = 6 C and 5 plKO (p = 0.9984). The density of total OL is increased in the cerebellar WM of plKO females compared to C littermates. **k**, Ratio of mature OLs (CC1 + cells) within the total OL lineage (Olig2 + cells) in the female cerebellar WM at P15. Data is presented as means ± SEM. Two-tailed unpaired Student’s t test with Welch’s correction (*p < 0.05). n = 6 C and 5plKO (p = 0.0225). A significantly reduction of OL maturation is observed in the female plKO as compared with littermate C.

**Extended Data Fig. 7:**
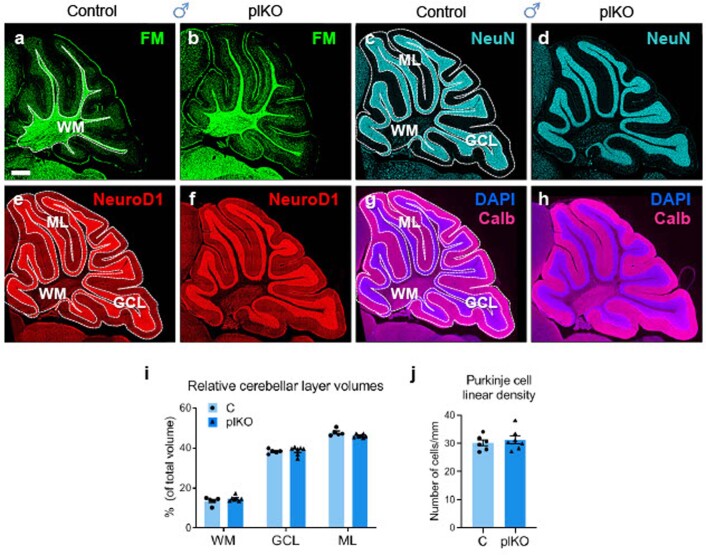
Anatomical analysis of cerebellar grey and white matters in C and plKO at P30. **a–h**, Cerebellar sagittal 40-μm-thick sections through the vermis in males. **a-b**, FluoroMyelin (FM) green staining. **c-d**, NeuN-immunofluorescence (marker for neuronal nuclei). **e-f**, NeuroD1-immunofluorescence (marker for granule cells). **g-h**, Calbindin-immunofluorescence (Purkinje cell marker; in magenta) and DAPI (in blue). **i**, Cerebellar layer volumes were unchanged in plKO when compared with C mice. Data is presented as means ± SEM from 6 sections/animal. Two-way ANOVA with Sidak’s multiple comparisons test. n = 5 C and 7plKO (WM: p = 0.48; GCL: p = 0.98; ML: p = 0.22). **j**, Purkinje cell linear density in the lobule VI-VII of C and plKO males at P30. Data is presented as means ± SEM from 6 sections/animal. Two-tailed unpaired Student’s t test with Welch’s correction. n = 6 C and 7plKO (p = 0.556). Calb, calbindin; GCL, granule cell layer; ML, molecular layer; WM, white matter. Scale bar, 200 μm.

We found that the density of OL lineage cells (Olig2^+^), and particularly mature OLs (CC1^+^), were significantly increased in the male plKO cerebellum at P30 compared to controls (Extended Data Fig. 6a,b). Increased OPC proliferation during fetal life is suggested by the results of co-labelling with BrdU at E15.5 and Olig2 expression at P30 (Extended Data Fig. 6c). Additionally, within the OL lineage, an acceleration of the maturation from PDGFRα^+^ OPCs to mature CC1^+^ OLs was observed in the plKO mice at P15 (Extended Data Fig. 6d–i). Interestingly, a sex-divergent progression of the OL lineage was observed at P15. At this age, whereas the density of Olig2^+^ cells was increased in plKO females in the same range as in plKO males (Extended Data Fig. 6d,j), the OL maturation (given by the CC1^+^/Olig2^+^ cell number ratio) was reduced (Extended Data Fig. 6h,k). Overall, these results are consistent with the ultrastructural analysis of myelin and indicate short-term, sex-independent (embryonic OPC proliferation) and long-term, sex-dependent (OL maturation) modifications of cerebellar OL lineage progression and OL maturation in mice lacking placental ALLO. No volumetric change in the different cerebellar layers (Extended Data Fig. 7a-i) nor Purkinje cell linear density (Extended Data Fig. 7j) were observed in the plKO mice.

### Placental ALLO insufficiency leads to autism spectrum disorder-like behaviors in males

**Extended Data Fig. 8:**
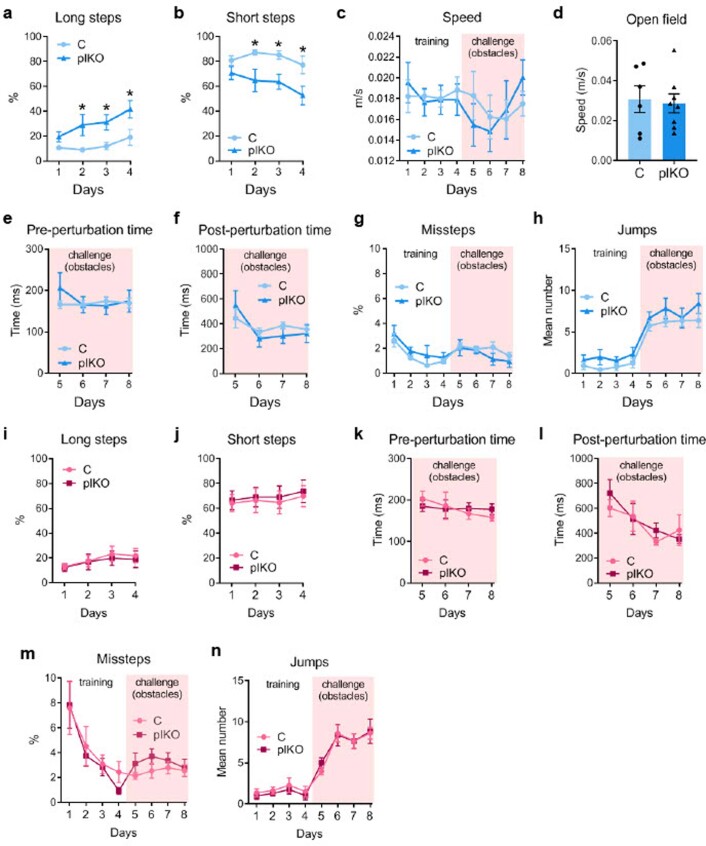
Locomotor assessment in the Erasmus ladder at P30. **a–h**, Performance on the Erasmus ladder. **a-b**, The analysis of baseline gait and coordination on days 1-4 showed significantly longer steps in adult male plKO mice than in their control littermates. Data is presented as means ± SEM. Two-way repeated-measures ANOVA with Sidak’s multiple comparisons test (*p < 0.05). n = 8 C and 6 plKO. Long steps=Day 1: p = 0.64; Day 2: p = 0.031; Day 3: p = 0.039; Day 4: p = 0.011. Short steps=Day 1: p = 0.61; Day 2: p = 0.029; Day 3: p = 0.039; Day 4: p = 0.015. **c**, Speed on the ladder was unchanged between C and plKO mice. Data is presented as means ± SEM. Two-way repeated-measures ANOVA with Sidak’s multiple comparisons test (n = 8 C and 6 plKO). **d**, Locomotor speed was unchanged in the open-field. Data is presented as means ± SEM. Two-tailed unpaired Student’s t test with Welch’s correction (n = 6 C and 8 plKO). **e-h**, The associative cerebellar learning was tested with the introduction of a conditioned tone and obstacle on days 5-8. The plKO mice exhibited no motor learning deficit. Data is presented as means ± SEM. Two-way repeated-measures ANOVA with Sidak’s multiple comparisons test (n = 8 C and 6plKO). **i-j**, The analysis of baseline gait and coordination on days 1-4 showed no difference in female plKO mice vs their control littermates. Data is presented as means ± SEM. Two-way repeated-measures ANOVA with Sidak’s multiple comparisons test (n = 7 C and 8plKO). **k-n**, The associative cerebellar learning was tested with the introduction of a conditioned tone and obstacle on days 5-8. The plKO mice exhibited no motor learning deficit. Data is presented as means ± SEM. Two-way repeated-measures ANOVA with Sidak’s multiple comparisons test (n = 7 C and 8 plKO).

Cerebellar circuits provide precise spatiotemporal control of sensorimotor behavior, and cerebellar contribution to cognitive functions has more recently been recognized. Thus, developmental disruption of cerebellar circuits, either through neuronal or glial alterations, might modify motor and cognitive performance. Cerebellar-dependent tests were first selected to assess locomotor and motor learning behaviors. Behavior testing on the Erasmus ladder, an advanced, fully automated system for comprehensive real-time monitoring of cerebellar-mediated motor function, was performed at P30, the earliest validated age^17^, to align with the anatomical analysis. At baseline, male plKO mice exhibited a mild alteration of their walking pattern, with higher percentage of long steps (Extended Data Fig. 8a,b). The frequency of missteps, often increased in ataxia models, was unchanged (Extended Data Fig. 8g). The locomotion speed was similar in male plKO and C on the Erasmus ladder, consistent with open field testing results (Extended Data Fig. 8c,d). An associative cerebellar learning task pairing a tone with a ladder obstacle revealed no cerebellar motor learning deficit in male plKO mice (Extended Data Fig. 8e,f). Female plKOs exhibited no locomotor alterations nor learning deficits (Extended Data Fig. 8i–n). Overall, our data suggest that plKO mice do not display major locomotor, ataxia-like or associative motor learning alterations. The only abnormal locomotion feature identified in plKO mice on the Erasmus ladder was an increased frequency of long steps by males. More frequent long steps or longer stride was previously observed in genetic mouse models with impeded intrinsic or synaptic Purkinje cell plasticity^18,19^. However, contrasting outcomes on the Erasmus ladder were also described in mice with related physiological impairments^20^. Future studies focusing on Purkinje cell physiology might elucidate the precise mechanisms underlying the subtle walking pattern abnormalities seen in plKO males. Interestingly, longer stride was also reported as an autism spectrum disorder (ASD)-associated feature in human and mouse^19,21^.

**Extended Data Fig. 9:**
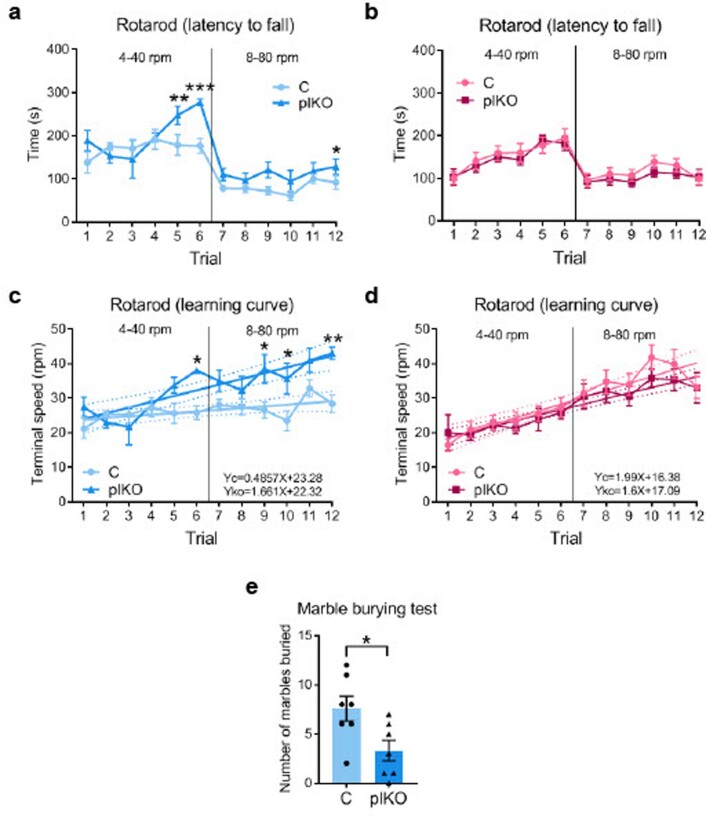
Locomotor and balance behavioral testing at P30. **a–d**, Accelerating rotarod performance at 4 to 40 rpm (trials 1-6) and 8 to 80 rpm (trials 7-12). **a-b**, Time to fall off is presented at 4 to 40 rpm and 8 to 80 rpm in males (a) and females (b). Data is presented as means ± SEM. Two-way repeated-measures ANOVA with Sidak’s multiple comparisons test (*p < 0.05, **p < 0.01, ***p < 0.005). Males: n = 9 C and 7plKO (Trial1: p = 0.4346; Trial2: p = 0.9961; Trial3: p = 0.9918; Trial4: p > 0.9999; Trial5: p = 0.0386; Trial6: p = 0.0016; Trial7: p = 0.9420; Trial8: p = 0.9995; Trial9: p = 0.4957; Trial10: p = 0.9118; Trial11: p = 0.9377; Trial12: p = 0.0495); Females: n = 6 C and 4 plKO (Any trial: p > 0.98). **c-d**, Terminal speed of rotation on the accelerating rotarod, presented at 4 to 40 rpm (trials 1-6) and 8 to 80 rpm (trials 7-12), was used to calculate learning rate (linear regression). The slopes from C and plKO are significantly different in males (c) (F = 11.32, DFd=20, p = 0.0031) but not in females (d). Data is presented as means ± SEM. Two-way repeated-measures ANOVA with Sidak’s multiple comparisons test (*p < 0.05, **p < 0.01, ***p < 0.005). Males: n = 9 C and 7 plKO (Trial1: p > 0.99; Trial2: p = 0.4176; Trial3: p = 0.0328; Trial4: p = 0.6343; Trial5: p = 0.9589; Trial6: p = 0.0328; Trial7: p = 0.6343; Trial8: p = 0.9589; Trial9: p = 0.0372; Trial10: p = 0.0328; Trial11: p = 0.4022; Trial12: p = 0.0045); Females: n = 6 C and 4 plKO (Any trial: p > 0.9). **e**, Marble burying test. Graph represents the number of marbles at least 2/3^rd^ buried over 30-min. Data is presented as means ± SEM. Two-tailed unpaired Student’s t test with Welch’s correction (*p < 0.05). n = 7 mice/group (p = 0.023).

To further evaluate balance, motor coordination and learning, mice were tested on an accelerating rotarod. Trained male, but not female, plKO mice displayed longer latency to fall and increased terminal speed (Extended Data Fig. 9a,b). The learning rate of individual mice, given by a linear regression analysis, was also enhanced in male plKO mice (Extended Data Fig. 9c,d). Greater learning in plKO males on the accelerating rotarod might be linked to their increased cerebellar FA, as previously described^22^. This gain of function, although rare, has been described in ASD mouse models, in which it has been attributed to increased stereotyped motor routine^23^. Although the underlying molecular mechanisms and circuits might differ between models, similar repetitive/stereotypical behavior could play a role in the enhanced rotarod learning displayed by plKO males.

**Fig. 3:**
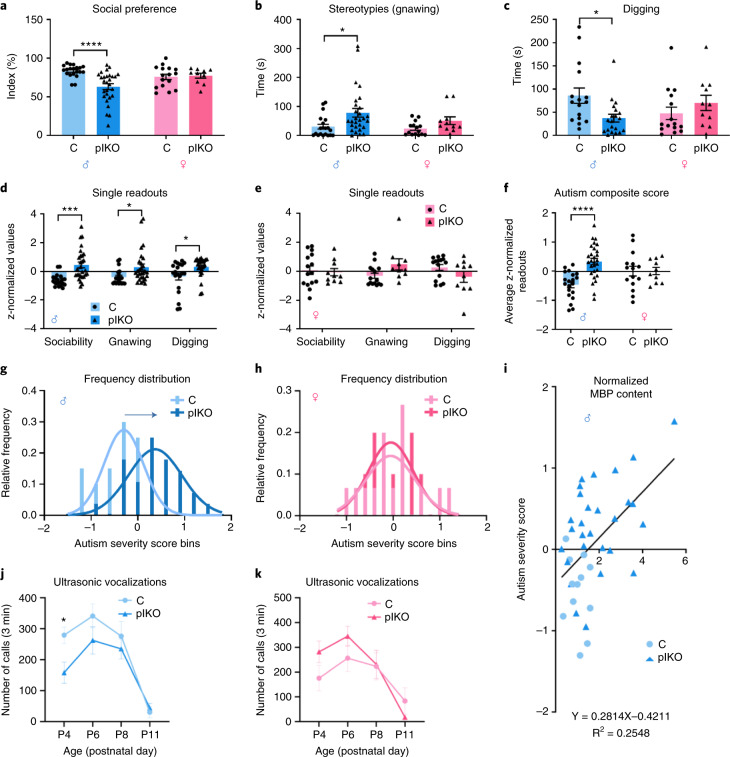
Male plKO mice exhibit ASD-like behavior. **a**, Three-chamber sociability test at P30. Data are presented as mean ± s.e.m. Two-way ANOVA with Sidak’s multiple comparison test (****P < 0.0001). Males: *n* = 20 C and 28 plKO (*P* = 0.0001); Females: *n* = 16 C and 10 plKO (*P* > 0.9999). **b**, Spontaneous gnawing time over 15 min at P30. Data are presented as mean ± s.e.m. Two-way ANOVA with Sidak’s multiple comparison test (*P < 0.01). Males: *n* = 20 C and 28 plKO (*P* = 0.0094); Females: *n* = 15 C and 11 plKO (*P* = 0.3982). **c**, Spontaneous digging time over 15 min at P30. Data are presented as mean ± s.e.m. Two-way ANOVA with Sidak’s multiple comparison test (*P < 0.05). Males: *n* = 20 C and 28 plKO (*P* = 0.0148); Females: *n* = 15 C and 11 plKO (*P* = 0.4781). **d**, z-standardized single significant behavior readouts that are integrated into the autism composite score in males at P30. Data are presented as mean ± s.e.m. Two-way ANOVA with Sidak’s multiple comparisons test (*P < 0.05; ***P < 0.0005). *n* = 20 C and 28 plKO. Sociability: *P* = 0.0002; Gnawing: *P* = 0.0239; Digging: *P* = 0.0301. **e**, z-standardized single significant behavior readouts that are integrated into the autism composite score in females at P30. Data are presented as mean ± s.e.m. Two-way ANOVA with Sidak’s multiple comparisons test. *n* = 15 C and 10 plKO. Sociability: *P* = 0.9802; Gnawing: *P* = 0.1419; Digging: *P* = 0.2979. **f**, ASD composite severity score at P30. Data are presented as mean ± s.e.m. Two-way ANOVA with Sidak’s multiple comparison test (****P < 0.0001). Males: *n* = 20 C and 28 plKO (*P* < 0.0001); Females: *n* = 16 C and 10 plKO (*P* > 0.9997). **g**,**h**, Relative frequency distribution of autism composite score bins at P30. Males: *n* = 20 C and 28 plKO; Females: *n* = 16 C and 10 plKO. **i**, Positive correlation between cerebellum MBP levels determined by western blot (normalized with GAPDH) and autism severity score in males at P30 (*n* = 12 C and 28 plKO). Linear regression. Deviation from zero: *P* = 0.0009. **j**,**k**, USVs at P4, P6, P8 and P11. Individual pups, males (**j**) and females (**k**), were separated from the dam and littermates, and their calls were recorded for 3 min. Data are presented as mean ± s.e.m. Two-way repeated measures ANOVA followed by Sidak’s multiple comparisons test (*P < 0.05). Males: *n* = 13 C and 15 plKO (P4: *P* = 0.0378; P6: *P* = 0.5714; P8: *P* = 0.9299; P11: *P* = 0.9609); Females: *n* = 7 C and 7 plKO (P4: *P* = 0.4587; P6: *P* = 0.6051; P8: *P* > 0.9999; P11: *P* = 0.7135). GAPDH, glyceraldehyde 3-phosphate dehydrogenase.

Beside its role in motor learning and coordination, the cerebellum contributes to cognitive processing and emotional control. Cerebellar impairments are often associated with ASD symptoms in humans and genetic mouse models^24,25^. Specifically, cerebellar WM abnormalities, including transient hyperplasia or dysregulation of myelin-related genes, were reported in patients with ASD^26,27^. We next asked whether mice lacking placental ALLO show autistic-like behavioral features by evaluating adult sociability and motor stereotypies, as well as isolation calling response at young ages. The three-chamber social behavior test revealed significant social interaction deficits in plKO males but not females (Fig. 3a). Stereotyped motor behavior was then investigated in spontaneously behaving mice. Male, but not female, plKO mice exhibited specific motor stereotypies, particularly increased gnawing (Fig. 3b). In addition, male plKO mice spent less time digging spontaneously or during a marble burying test (Fig. 3c and Extended Data Fig. 9e). Difficulties in social interactions and motor stereotypies are two ASD hallmarks, especially in males^28^. In addition, both increased and decreased digging and marble burying were noted as secondary ASD-like features related to emotional state and anxiety in several genetic ASD mouse models^19,21,29–31^.

Behavioral sex-linked differences between C and plKO mice can be detected even earlier in postnatal development. The ultrasonic vocalizations (USVs) emitted by the pups when separated from the dam (referred to as isolation calling response) were evaluated as a measure of the degree of aversive affective state and early communicative behavior. We found that plKO males produced fewer USVs than their littermate controls when separated from the dam (Fig. 3j). By contrast, no significant change was evidenced in the plKO females (Fig. 3k). Deficient isolation calling response has been detected in different mouse models of ASD^32^, indicating that insufficient provision of placental ALLO to the fetal brain can lead to a wide array of autistic-like behaviors in the male progeny.

### Cerebellar MBP correlates with ASD-like symptom severity

We calculated an autism severity composite index based on previously established scoring systems^33^. Behaviors were z-standardized with higher values representing higher symptom severity. Individual behavioral scores were significantly higher in plKO males compared to C but not in females (Fig. 3d,e). Thus, the combined z-scores yielded an elevated autism severity score in males (Fig. 3f). The relative frequency distribution of individual autism severity scores followed Gaussian distribution curves that differed in plKO males, which shifted to the right along the *x* axis (Fig. 3g,h). Interestingly, some of the plKO mice exhibited scores similar to control mice, suggesting a spectrum of behavioral changes in male plKOs. This variability might arise from natural variations in placental progesterone and ALLO levels, variable deletion of *akr1c14*, mixed genetic background of the mice or subtle changes in hormone exposure due to in utero position of the fetus.

A significant positive correlation between autism symptom severity scores and cerebellar MBP levels was seen in plKO males (Fig. 3i). This finding is of particular interest knowing that transient WM hyperplasia, myelin thickening and/or over-expression of OL lineage and myelin markers such as *olig2*, *mbp* and *mag* have been documented in the cerebellum of patients with ASD^26,27^ and several genetic ASD-like mouse models^34,35^. Together with the enhanced FA in the cerebellum-related tracts described above (Extended Data Fig. 4a), this correlation could reflect a short-range hyperconnectivity and/or a transient WM hyperplasia as seen in patients with autism^26,36^. The abnormal maturation speed of the OL lineage in plKO mice suggests dynamic, rather than uniform, changes of WM development, similar to the pattern seen across the lifespan of patients with ASD^26^. Overall, our placental mouse model recapitulates several characteristics of human studies, including the male greater vulnerability to perinatal brain injuries and ASD^19,23,27,37^.

### ALLO or GABA_A_ agonist rescues cerebellar and behavioral impairments

**Fig. 4:**
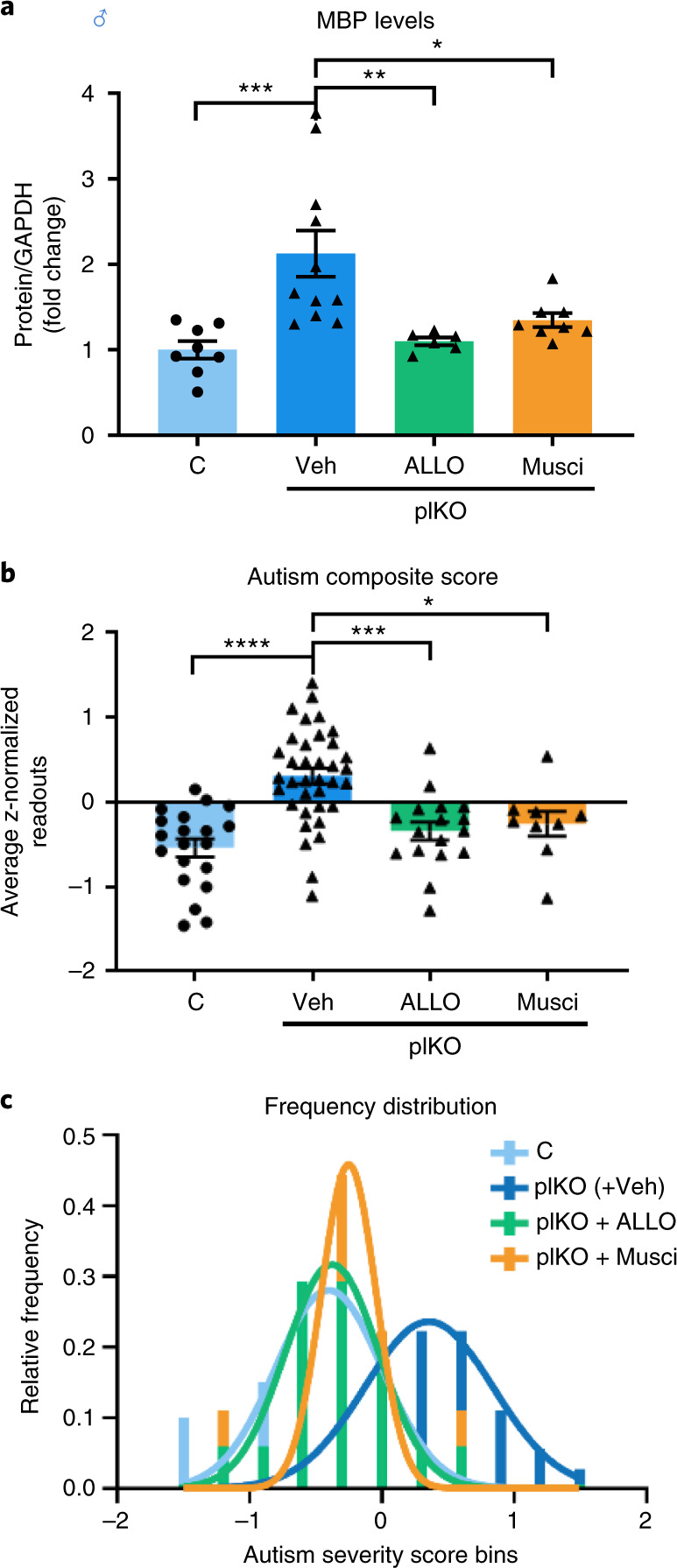
ALLO or muscimol administration during late gestation rescues MBP expression and abnormal behaviors in plKO males at P30. **a**, Western blot determination of MBP contents in the cerebellum at P30. Normalized data to GAPDH contents are presented as mean fold changes ± s.e.m. Dams received an intraperitoneal injection of ALLO (10 mg kg^−1^) or muscimol (Musci; 1 mg kg^−1^) at E15.5. One-way ANOVA with Tukey’s multiple comparisons test (*P < 0.05; **P < 0.01; ***P < 0.005). *n* = 8 C, 11 plKO+Veh, 6 plKO+ALLO and 8 plKO+muscimol. Comparisons: C versus plKO+Veh: *P* = 0.009; C versus plKO+ALLO: *P* = 0.988; C versus plKO+muscimol: *P* = 0.607; plKO+Veh versus plKO+ALLO: *P* = 0.0058; plKO+Veh versus plKO+muscimol: *P* = 0.027; plKO+ALLO versus plKO+muscimol: *P* = 0.8436. **b**,**c**, ASD composite score and frequency distribution of autism severity score bins at P30. plKO and plKO+Veh mice were combined because there was no significant difference between both groups (named plKO(+Veh)). One-way ANOVA with Tukey’s multiple comparisons test (***P < 0.005; ****P < 0.0005). *n* = 20 C, 3 6plKO(+Veh), 17 plKO+ALLO and 9 plKO+muscimol. Comparisons: C versus plKO(+Veh): *P* < 0.0001; C versus plKO+ALLO: *P* = 0.6226; C versus plKO+muscimol: *P* = 0.4933; plKO(+Veh) versus plKO+ALLO: *P* = 0.0002; plKO(+Veh) versus plKO+muscimol: *P* = 0.0187; plKO+ALLO versus plKO+muscimol: *P* = 0.9765. GAPDH, glyceraldehyde 3-phosphate dehydrogenase.

**Extended Data Fig. 10:**
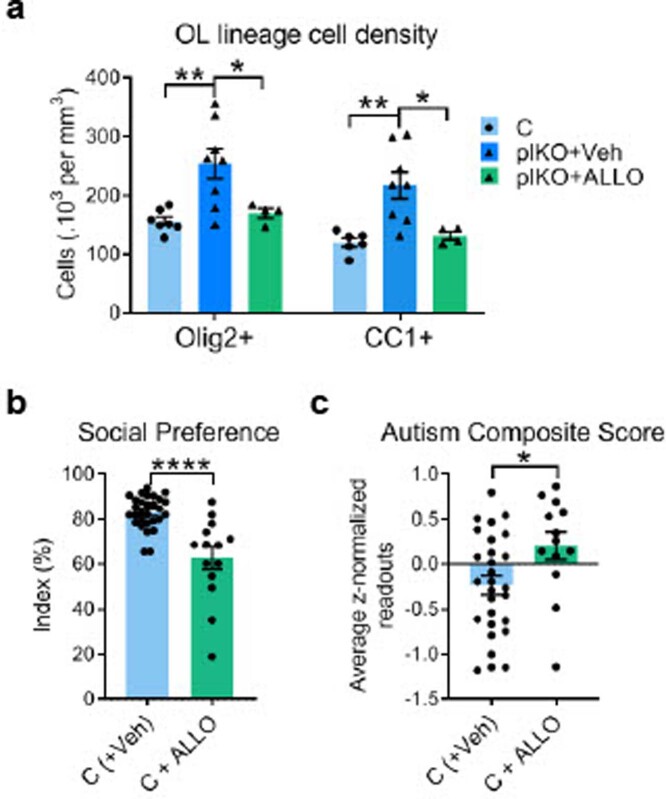
Cerebellar WM and behavioral impairments in male plKO mice can be prevented by pharmacological treatments at E15.5. **a**, Quantification of Olig2+ and CC1 + cell densities in the male cerebellar WM (n = 7 C, 8 plKO+Veh and 4 plKO+ALLO). Data presented as means ± SEM. Two-way ANOVA with Sidak’s multiple comparisons test (*p < 0.05; **p < 0.01). Olig2 + = C vs plKO+Veh: p = 0.0024; C vs plKO+ALLO: p = 0.9982; plKO+Veh vs plKO+ALLO: p = 0.041. CC1 + : C vs plKO+Veh: p = 0.0014; C vs plKO+ALLO: p = 0.9995; plKO+Veh vs plKO+ALLO: p = 0.0354. **b**, 3-chamber sociability test. Data is presented as means ± SEM. Two-tailed unpaired Student’s t test with Welch’s correction (****p < 0.001). n = 29 C(+Veh) and 14 C + ALLO (p < 0.0001). **c**, ASD composite severity score. Data presented as means ± SEM. Two-tailed unpaired Student’s t test with Welch’s correction (*p < 0.05). n = 28 C(+Veh) and 13 C + ALLO (p = 0.0261).

The therapeutic potential of ALLO to rescue cerebellar WM and behavioral abnormalities was then tested by administering ALLO (10 mg kg^−1^) or vehicle (sesame oil) to dams carrying mixed litters at E15.5, the period of maximal difference of ALLO levels in the plKO mice compared to C. A single injection of ALLO during gestation prevented the cerebellar MBP up-regulation (Fig. 4a), normalized OL density in the cerebellar WM (Extended Data Fig. 10a) and reduced the autism severity score (Fig. 4b,c). To assess the specific contribution of GABA_A_R signaling to this rescue, muscimol (1 mg kg^−1^), a selective GABA_A_ agonist, was administered instead of ALLO. Muscimol injection resulted in similar molecular and behavioral rescue in plKO males (Fig. 4a-c), although the contribution of other minor, GABA_A_-independent ALLO actions cannot be completely excluded^38–40^. Interestingly, a fraction of C mice exposed to ALLO treatment exhibited decreased social preference and subsequent higher autism score (Extended Data Fig. 10b,c), suggesting that excess ALLO exposure during fetal life might be detrimental. Interestingly, increased PROG exposure, along with other steroids, was previously linked to ASD risk in boys^41^. Our results support the potential therapeutic utility of ALLO administration during gestation if ALLO or its precursors are determined to be low (as might occur with chronic placental insufficiency) but, additionally, suggest the need to maintain fetal ALLO exposure within an appropriate physiological window.

### Myelin genes are similarly altered in preterm infants and plKO mice

**Fig. 5:**
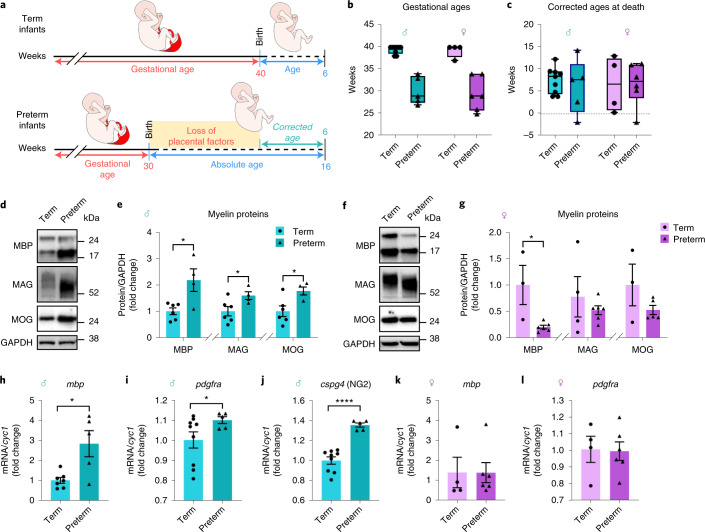
Myelin proteins are dysregulated in a sex-linked manner in the cerebellar vermis of preterm infants. **a**, Schematic of the age terminology during the perinatal period used for the human study. **b**, Distribution of donors’ gestational age at birth. **c**, Distribution of donors’ corrected age at death. Graphs in **b** and **c** show box and whisker plots (including minima, maxima and median values) with single values. **d**,**e**, Western blot analysis of myelin-related proteins in the cerebellar vermis of term and preterm male infants at an average corrected age of 6 weeks. Data are presented as mean fold changes ± s.e.m. (*n* = 6 T and 4 PT). Multiple unpaired *t*-tests with Holm–Sidak multiple comparison test (*P < 0.05). MBP: *P* = 0.014; MAG: *P* = 0.044; MOG: *P* = 0.024. **f**,**g**, Western blot analysis of myelin-related proteins in the cerebellar vermis of term and preterm female infants at an average corrected age of 6 weeks. Data are presented as mean fold changes ± s.e.m. Multiple unpaired *t*-tests with Holm–Sidak multiple comparison test (*P < 0.05). MBP: *n* = 3 T and 6 PT (*P* = 0.015); MAG: *n* = 3 T and 6 PT (*P* = 0.169); MOG: *n* = 3 T and 5 PT (*P* = 0.179). **h**–**j**. qRT–PCR for myelin- and OL-related genes normalized to *cyc1* in male infants. Data are presented as mean fold changes ± s.e.m. Two-tailed unpaired Student’s *t*-test with Welch’s correction (*P < 0.05, ****P < 0.0001). *mbp*: *n* = 7 T and 5 PT (*P* = 0.046); *pdgfra:* *n* = 9 T and 5 PT (*P* = 0.0482); c*spg4:* *n* = 9 T and 5 PT (*P* < 0.0001). **k**,**l**, qRT–PCR for myelin- and OL-related genes normalized to *cyc1* in female infants. Data are presented as mean fold changes ± s.e.m. Two-tailed unpaired Student’s *t*-test with Welch’s correction. *mbp*: *n* = 4 T and 6 PT (*P* = 0.9923); *pdgfra:* *n* = 4 T and 6 PT (*P* = 0.9122). *Cspg4*, chondroitin sulfate proteoglycan 4 gene; *Cyc1*, cytochrome C1 gene; GAPDH, glyceraldehyde 3-phosphate dehydrogenase; *Pdgfra*, platelet-derived growth factor receptor alpha gene. PT, preterm; T, term. Source data

To further evaluate the translational potential of our findings, we asked whether preterm birth, a condition characterized by premature loss of placenta and a risk factor for ASD^42^, was associated with cerebellar myelin alterations similar to those seen in plKO mice. We examined 24 postmortem cerebellar vermises from term (>37 weeks of gestation) and preterm (<34 weeks of gestation) infants who died at a mean corrected age of 6 weeks (Fig. 5a–c and Supplementary Table 3). Compelling similarities between our mouse model and human pathology were found in cerebellar MBP expression in preterm males and females (Fig. 5d–h,k) compared to corrected age-matched specimens. Furthermore, the migration downshift of MAG on western blot gels was observed in human preterm male cerebellum, similar to our findings in plKO males (Figs. 2a and 5d), again suggesting accelerated maturation of myelin^10^. The higher levels of *pdgfra*- and *cspg4*-mRNA in the cerebellum of preterm males also suggest increased OPC proliferation (Fig. 5i,j,l), as temporarily seen in the plKO males (Extended Data Fig. 6e). This striking correlation between mice exposed to ALLO insufficiency and infants born preterm suggests that altered ALLO exposure might contribute to abnormal preterm brain development.

## Discussion

Our study empirically defines a critical role for a specific placental hormone that can alter fetal brain development in late gestation with postnatal developmental consequences. Many neuroactive hormones are produced in the placenta in late gestation and might affect specific stages of neurogenesis and gliogenesis, neuronal migration and circuit formation. We focused on ALLO because this placental steroid hormone normally peaks in the second half of gestation, when placental insufficiency becomes evident and when preterm birth occurs. Interestingly, in severe pre-eclampsia, a pregnancy condition associated with both preterm birth and ASD, plasma ALLO levels in pregnant women are reduced, as compared to control group^43^, strengthening the potential link among ALLO, prematurity and ASD risk.

Many brain regions have been linked to autism, but structural and functional cerebellar abnormalities are among the most consistent findings^44–46^. Early cerebellar injury or dysfunction carries a high relative risk (up to 36×) for ASD, exceeded only by genetic factors^25^. Both neuronal dysfunction, especially of cerebellar Purkinje cells, and WM changes have been linked to ASD phenotypes. Cerebellar transcriptome analysis highlighted WM-associated pathways as a top category of altered genes. Anatomical myelination changes, consistent with these transcriptomic findings, suggest that oligodendrogenesis and myelination significantly contribute to the observed outcomes. In our mouse model, Purkinje cell number appears normal (Extended Data Fig. 7j), but the contribution of Purkinje cell physiology to the myelin and behavioral phenotypes will be assessed in future studies. Additionally, the local specificity of changes will need to be further assessed. Motor and non-motor functions of the cerebellum are controlled by specific subdivisions—that is, lobules and modules—that are characterized by specific inputs and outputs, Purkinje cell firing frequency, degree of myelination and neurochemical signatures (for example, zebrin expression)^47^. Future studies addressing whether the increased myelin thickness in the cerebellum of plKO mice is global or restricted to functional zones, and whether it correlates with Purkinje cell firing frequency, might elucidate how placental ALLO insufficiency alters specific cerebellar functions that are linked to ASD.

Our experiments demonstrate a treatment-reversible link between specific placental ALLO insufficiency and sex-specific cerebellar myelination associated with an ASD-like phenotype. It is well documented that female and male brains might take different developmental trajectories in physiological and pathological conditions^48^, but there is still limited mechanistic information on the underlying causes of sex differences^49^. The results we describe here suggest that, in the case of ALLO insufficiency and cerebellar myelination, this sex-linked divergence occurs primarily in the postnatal period. Furthermore, the sex-linked phenotypes of our mouse model parallel human pathological features, strengthening the translational value of our findings. These experiments suggest that the cerebellum is the primary brain region affected by the lack of placental ALLO in males. Other brain regions might be altered specifically in females, as our transcriptomic assessment suggests (Extended Data Fig. 5). Undoubtedly, such complex sexual dimorphism builds upon the interplay of a myriad of factors throughout prenatal and postnatal development. Further investigation of brain region-specific, sex-linked trajectories under normal and pathological conditions can pave the way for personalized risk assessment, diagnosis and treatment informed by patient sex and hormone levels.

Increased risk of ASD diagnosis after extremely preterm birth^50^, particularly in males, suggests that loss of placental hormones might contribute to specific human neurobehavioral outcomes in previously unanticipated ways. Our findings lay the groundwork for developing hormone replacement strategies to normalize the developmental milieu and prevent long-term impairments in neurobehavior.

## Methods

### Mice

All the procedures on experimental animals were performed in accordance with the protocol approved by the Institutional Animal Care and Use Committee at Children’s National Medical Center (protocol no. 30534 (PI: Penn)) and at Columbia University Medical Center (protocol nos. AC-AABE 6553 (PI: Penn) and AC-AABF 5550 (PI: Yang)). Mice were housed on a 12-h light/dark cycle and had ad libitum access to food and water.

The akr1c14-floxed mouse was constructed with inGenious Targeting Laboratories. The construct targeted a 3.15-kb region that includes exons 7–9 of *akr1c14*, with a 5′ homology arm extending to the position of a LoxP-FRT flanked Neo cassette and a 3′ homology arm extending to a single LoxP site just 5′ of exon 10 (Fig. 1b). The targeting vector was electroporated into embryonic stem cells, which were cloned and screened. Confirmed clones were micro-injected into C57Bl/6 blastocysts. The Neo cassette was later removed using flipase. The resulting mice are designated as the *akr1c14*-floxed line. akr1c14^fl/wt^ and akr1c14^fl/fl^ mice appear phenotypically normal. The final placental 3α-HSD knockout was generated by successive crosses of progenitor akr1c14-floxed mice and Cyp19a-Cre mice (generous gift of G. Leone), which express Cre exclusively in placental trophoblast cells^6^. Mouse, unlike human, does not express endogenous placental Cyp19a, which codes for aromatase, but the human Cyp19a promotor effectively drives placenta-specific Cre expression^6^. Cyp19a-Cre females were crossed to homozygous akr1c14^fl/fl^ males. Female offspring of these crosses that were positive for Cre with two floxed alleles were used as the dams of experimental mice. These akr1c14^fl/fl^ females carrying Cyp19a-Cre were crossed to akr1c14^fl/fl^ males, so that half of resulting offspring are both homozygous akr1c14^fl/fl^ and positive for placental Cre. In tests of recombination specificity (Fig. 1e), the presence of both LoxP sites (indicating un-recombined DNA) was ascertained using a primer set, including a forward primer homologous to a region 5′ of exon 7, and a reverse primer incorporating the 3′ LoxP site between exons 9 and 10. This primer pair was specific to the floxed allele due to the inclusion of the LoxP site in the reverse primer sequence. It did not amplify the wild-type allele. The presence of the recombined LoxP site was ascertained using a primer set, which flanks exon 10 such that the recombined allele (164 bp in length) amplifies, whereas the intact allele is too long to amplify (3,396 bp). These mice in which *akr1c14* is deleted specifically in the placenta are akr1c14^Cyp19a−/−^, designated as akr1c14^Cyp19a^KO (plKO) for simplicity. An additional half of offspring are negative for placental Cre and designated as controls (C).

Cyp19a-Cre transgenic mice were crossed with ROSA26-YFP mice (The Jackson Laboratory, no. 006148) to obtain Cyp19a-Cre:R26R-EYFP littermates.

### Human samples

Human cerebellar vermes were obtained from the National Institutes of Health (NIH) NeuroBioBank (request ID no. 709). Donors consisted of 6-week-old term infants (average age) and corrected age-matched preterm infants, excluding those with major congenital anomalies or known genetic diagnoses and those with meningitis or stroke as cause of death. Sex, gestational weeks, absolute age (postnatal age) and corrected age are shown in Supplementary Table 3. Frozen tissues were preserved at –80 °C.

### Drug injections

Pregnant dams received one intraperitoneal injection of allopregnanolone (Tocris Bioscience, no. 3653) diluted in sesame oil (vehicle; Sigma-Aldrich, no. S3547) at E15.5 at a dose of 10 mg kg^−1^ of body weight. Muscimol (Tocris, no. 0289) was diluted in saline solution and injected intraperitoneally in pregnant mice at E15.5 (1 mg kg^−1^). Allopregnanolone and muscimol injections were done in the morning, during sleep time, to limit the effect of the drug-induced sedation on dams’ activity. Doses were chosen based on prior pharmacological studies in mice^51,52^, and the timing was an empirical choice based on *akr1c14* gene expression peak at E14.5 (Fig. 1a). BrdU (50 mg kg^−1^) dissolved in saline solution was injected intraperitoneally in dams at E15.5.

### Mass spectrometry

#### Steroid extraction

Pregnenolone (PREG), PROG, ALLO, epiallopregnanolone (EPIALLO), ALLOTHDOC and 3α5α-tetrahydrotestosterone (3α5α-THT) were identified and quantified simultaneously in individual tissues by gas chromatography–tandem mass spectrometry (GC–MS/MS) as previously described^53^. Placental (65–185 mg) and fetal brain (61–130 mg) tissues of male and female wild-type and plKO mice at E17.5 were weighed and stored at −20 °C until GC–MS/MS analysis. Briefly, steroids were first extracted from placentas, and brains with 10 volumes of methanol (MeOH) and the following internal standards were added to the extracts for steroid quantification: 2 ng of epietiocholanolone for PREG, ALLO, EPIALLO, ALLOTHDOC and 3α5α-THT and 2 ng of ^13^C_3_-PROG for PROG. Samples were purified and fractionated by solid-phase extraction with the recycling procedure^54^. The extracts were dissolved in 1 ml of MeOH and applied to the C18 cartridge (500 mg, 6 ml, International Sorbent Technology), followed by 5 ml of MeOH/H_2_O (85/15, vol/vol). The flow-through, containing the free steroids, was collected and dried. After a previous re-conditioning of the same cartridge with 5 ml of H_2_O, the dried samples were dissolved in MeOH/H_2_O (2/8, vol/vol) and re-applied. The cartridge was then washed with 5 ml of H_2_O and 5 ml of MeOH/H_2_O (1/1, vol/vol), and unconjugated steroids were eluted with 5 ml of MeOH/H_2_O (9/1, vol/vol). The fraction containing the unconjugated steroids was then filtered and further purified by high-performance liquid chromatography (HPLC). The HPLC system is composed of a WPS-3000SL analytical autosampler and an LPG-3400SD quaternary pump gradient coupled with a SR-3000 fraction collector (Thermo Fisher Scientific). The HPLC separation was achieved with a LiChrosorb Diol column (25 cm, 4.6 mm, 5 μm) in a thermostated block at 30 °C. The column was equilibrated in a solvent system of 90% heptane and 10% of a mixture composed of heptane/isopropanol (85/15, vol/vol). Elution was performed at a flow rate of 1 ml min^−1^, first 90% heptane and 10% of heptane/isopropanol (85/15, vol/vol) for 15 min, and then with a linear gradient to 100% acetone in 2 min. The column was washed with acetone for 15 min. Steroids were collected in the time range of 15–29 min and were derivatized with 25 μl of HFBA and 25 μl of anhydrous acetone for 1 h at 20 °C. Samples were dried under a stream of nitrogen and resuspended in heptane.

#### GC–MS/MS analysis

GC–MS/MS analysis of the biological extracts was performed using an AI 1310 autosampler, a Trace 1310 gas chromatograph and a TSQ 8000 mass spectrometer (Thermo Fisher Scientific). Injection was performed in the splitless mode at 250 °C (1 min of splitless time), and the temperature of the gas chromatograph oven was initially maintained at 80 °C for 1 min and ramped between 80 °C and 200 °C at 20 °C min^−1^ and then ramped to 300 °C at 5 °C min^−1^ and finally ramped to 350 °C at 30 °C min^−1^. The helium carrier gas flow was maintained constant at 1 ml min^−1^ during the analysis. The transfer line and ionization chamber temperatures were 300 °C and 200 °C, respectively. Electron impact ionization was used for mass spectrometry with ionization energy of 70 eV, and GC–MS/MS analysis was performed in multiple reaction monitoring mode with argon as the collision gas. GC–MS/MS signals were evaluated using a computer workstation by means of the software Excalibur, release 3.0 (Thermo Fisher Scientific). Identification of steroids was supported by their retention time and two or three transitions. Quantification was performed according to the more abundant transition with a previously established calibration curve. The range of the limit of detection was roughly 0.5–20 pg according to the steroid structure. The GC–MS/MS analytical procedure was fully validated in terms of accuracy, reproducibility and linearity in mouse brain^53^.

### 3′ mRNA sequencing

Tissue collection and RNA extraction: Cerebellum, bilateral cerebral cortices and bilateral hippocampi from C and plKO mice (males and females) at P30 (three animals per group) were dissected and flash-frozen. Tissue homogenization and total RNA extraction were performed using the *mir*Vana Isolation Kit (Thermo Fisher Scientific, no. AM1560) according to the manufacturer’s instructions.

#### Library preparation and sequencing for mRNA

The cDNA libraries were prepared using the QuantSeq 3′ mRNA-Seq Library Prep Kit FWD for Illumina (Lexogen) as per the manufacturer’s instructions. Briefly, total RNA was reverse transcribed using oligo (dT) primers. The second cDNA strand was synthesized by random priming, in a manner that DNA polymerase is efficiently stopped when reaching the next hybridized random primer; therefore, only the fragment closed to the 3′ end was captured for indexed adapter ligation and PCR amplification. The processed libraries were assessed for its size distribution and concentration using BioAnalyzer High Sensitivity DNA Kit (Agilent Technologies, no. 5067-4626). Pooled libraries were diluted to 2 nM in EB buffer (Qiagen, no. 19086) and then denatured using the Illumina protocol. The libraries were pooled and diluted to 3 nM using 10 mM Tris-HCl, pH 8.5, and then denatured using the Illumina protocol. The denatured libraries were diluted to 10 pM by pre-chilled hybridization buffer and loaded onto a TruSeq v2 Rapid flow cell on an Illumina HiSeq 2500 and run for 50 cycles using a single-read recipe according to the manufacturer’s instructions. De-multiplexed sequencing reads were generated using Illumina bcl2fastq (release version 2.18.0.12) allowing no mismatches in the index read.

#### Data analysis

After the quality and polyA trimming by BBDuk^55^ and alignment by HISAT2 (version 2.1.0)^56^, read counts were calculated using HTSeq^57^ by supplementing Ensembl gene annotation (GRCm38.78). DE analysis and MA plots were done using TCC R package (version 1.12.1, https://bioconductor.riken.jp/packages/3.3/bioc/manuals/TCC/man/TCC.pdf)^58^. After getting DEGs, hierarchical clustering of DEGs as a heat map was performed using Partek Genomics Suite (version 6.6, http://www.partek.com/partek-genomics-suite/) (Partek). No outlier was identified in our biological replicates, as indicated by principal component analysis and scatter matrices. DEGs met the following guidelines: *P* < 0.05; fragments per kilobase of transcript per million mapped reads values >1; and fold change > 1.5. The limits of these cutoffs were validated by RT–PCR on samples from other mouse cohorts. IPA was used to identify the top biological functions and disease processes that were differentially regulated. This software is based on the Ingenuity Pathway Knowledge Base for genetic interaction, which derives from the scientific literature, each network connection being supported by previous publications^59^. To determine and visualize the degree of gene overlaps in datasets, Venn analysis was performed using Venny 2.1 (http://bioinfogp.cnb.csic.es/tools/venny/).

### RT–PCR

Tissues were homogenized in TRIzol Reagent (Thermo Fisher Scientific); total RNA was extracted with the RNeasy Mini Kit (Qiagen, no. 74104). Next, 1 µg of RNA was used to make cDNA with the iScript cDNA Synthesis Kit (Bio-Rad, no. 1708891). All primer pairs were designed and validated in-house for efficiency and specificity. RT–PCR experiments were performed on cDNA samples in the presence of PowerUp SYBR Green Master Mix (Thermo Fisher Scientific, no. 1725271) with specific primers at 100 nM using the CFX96 Touch Real-Time PCR Detection System (Bio-Rad). The cDNA-generated signals for target genes were internally corrected with phosphoglycerate kinase 1 (*pgk1*) in mouse postnatal brains, tyrosine 3-monooxygenase/tryptophan 5-monooxygenase activation protein zeta (*ywhaz)* in mouse placentas and embryonic brains or cytochrome C1 (*cyc1*) in human tissues. The regulation fold changes were determined with the 2^−∆∆Cq^ method^60^.

### MRI

#### Brain preparation

Brains left within the skull were prepared for MRI using the following method. Mice were anesthetized and intracardially perfused at a rate of 1 ml min^−1^, with 30 ml of 0.1 M PBS containing 10 U/100 ml heparin (Sigma-Aldrich) and 2 mM ProHance (gadolinium contrast agent, Bracco Diagnostics) followed by 30 ml of 4% paraformaldehyde (PFA) and 2 mM ProHance. After perfusion, the mice were decapitated, and the skin and the lower jaw were removed. Brains in skulls were post-fixed in 4% PFA and 2 mM ProHance at 4 °C overnight and then kept in 0.1 M PBS containing 2 mM ProHance and 0.02% sodium azide for at least 1 month before MRI scanning^61,62^.

#### DTI acquisition

A multi-channel 7-Tesla MRI scanner (Agilent) was used to image the brains. For DTI Imaging, 12 brains were scanned in parallel using a 3D diffusion-weighted fast spin echo scan^63,64^. Parameters for the DTI sequence were as follows: TR of 270 ms, echo train length of 6, first TE of 32 ms and a TE of 10 ms for the remaining five echoes, 1 average, field of view of 14 mm ×14 mm × 25 mm and a matrix size of 180 × 180 × 324, resulting in an image with 78-µm isotropic voxels. Five *b* = 0 s mm^−^^2^ images and 30 high *b*-value (*b* = 2,147 s mm^−^^2^) images in 30 different directions were acquired, using the Jones30 scheme^65^. Total imaging time was approximately 12 h. The FSL software package (FMRIB) was used to generate FA and mean diffusivity (MD) maps for each brain sample.

#### MRI registration and analysis

To assess any changes to the mouse brains due to genotype and sex, the *b* = 0 s mm^−^^2^ images were registered linearly (6 followed by 12 parameter) and non-linearly together. A combination of mni_autoreg tools^66^ and ANTs (advanced normalization tools)^67^ were used to perform the registrations. A population atlas, representing the average anatomy of the study samples, was created when all scans were resampled with an appropriate transform. The final registration results are the individual images deformed into alignment with one another in an unbiased manner. The analysis of the individual DTI parameters compare intensity differences of FA and MD between genotype and sex. Warping an available classified MRI mouse brain atlas onto the population average allows for significant differences in the diffusion measures of segmented structures to be calculated^68–71^.

### Histology

#### Section preparation and staining

Animals were anesthetized using isoflurane (Isothesia, Henry Schein Animal Health) and transcardially perfused with 20 ml of 1× PBS followed by 30 ml of 4% PFA, as previously described. Brains were post-fixed for 24 h in 4% PFA and cryoprotected in 20% sucrose in PBS. Serial 40-μm-thick sagittal brain/cerebellar sections were then collected using a sliding microtome. Placentas were collected at E17.5 after deep anaesthesia of the dams, drop-fixed in 4% PFA and cryoprotected in 30% sucrose. Frozen 20-μm cross-sections were obtained with a cryostat. Immunohistochemistry on brain or placenta sections was performed using the following antibodies: rabbit anti-MBP (Abcam, no. ab40390, 1:500), mouse anti-APC clone CC1 (EMD Millipore, no. MABC20, 1:500), rabbit anti-Olig2 (Abcam, no. ab9610, 1:500), mouse anti-neuronal nuclei (NeuN) (Millipore, no. MAB377, 1:500), goat anti-NeuroD1 (RD Systems, no. AF2746, 1:500), rabbit anti-calbindin (Swant, no. CB38, 1:1,000), rat anti-mouse PDGFRα (CD140a; BD Biosciences, 17-1401-81, 1:500) and chicken anti-GFP (Abcam, no. ab13970, 1:500). Sections were incubated with primary antibodies overnight at 4 °C containing 0.3% Triton and 10% normal donkey serum. Sections were then incubated with secondary antibodies (1:500) together with DAPI (Invitrogen, no. D1306, 1:1,000) for 2 h at room temperature. FluoroMyelin Green (Invitrogen, F34651, 1:500) was also added at this time for those sections. Secondary antibodies were used as follows: donkey anti-mouse Alexa-488 (Invitrogen, A-21202), donkey anti-rabbit Alexa-488 (Invitrogen, A-21206), donkey anti-mouse Alexa-555 (Invitrogen, A-31570), donkey anti-rabbit Alexa-555 (Invitrogen, A-31572), donkey anti-mouse Alexa-647 (Invitrogen, A-31571) and donkey anti-rabbit Alexa-647 (Invitrogen, A-31573). Finally, floating sections were mounted and cover-slipped using ProLong Gold Antifade Mountant (Thermo Fisher Scientific, P36930) before imaging.

#### In situ hybridization

For analysis of *akr1c14*-mRNA, E17.5 placental tissue was drop-fixed overnight in 4% PFA and then transferred through a sucrose gradient of 10%, 20% and 30% over 3 d. Tissue was cross-sectioned at 12 µm and assayed for *akr1c14*-mRNA using the Advanced Cell Diagnostics RNAScope system and an ACD probe designed to target the 621–1,989 region of *akr1c14* (NM_134072.1).

#### Fluorescence imaging and cell counting

Placenta cross-sections and whole brain or cerebellum sagittal sections were imaged using a virtual slide microscope using cellSens version 2.3 (VS 120, Olympus Life Science) under a ×20 objective. High-magnification images were taken under a confocal microscope with LAS X Life Science Microscope software version 2.7 (TCS SP8, Leica Microsystems). z-stack images were acquired with a step size of 1.55 μm and viewed using NIH ImageJ 1.53c (http://imagej.nih.gov/ij). The different cerebellar layers were delineated manually using the freehand selection tool in ImageJ. The density of Olig2-, CC1-, PDGFRα- and BrdU-positive cells was estimated using Stereo Investigator (MBF Bioscience). After delineating the cerebellar WM using the freehand selection tool on corresponding DAPI images, immunopositive cells were manually counted under the ×40 objective (Zeiss Axio Imager M2) using the optical fractional as a probe in grids randomly distributed on and covering 30% of the cerebellar WM. Cell densities were then calculated by using the individual WM area values and the mean measured section thickness. The linear density of Purkinje cells (Purkinje cells per millimeter) was determined in the lobule VI–VII by drawing a freehand line through the center of the cell bodies.

### Electron microscopy

#### Sample preparation

P30 mice were anesthetized with a 10:1 mixture of ketamine/xylazine in saline vehicle such that each mouse receives 100 mg kg^−1^ of ketamine and 10 mg kg^−1^ of xylazine, respectively. Under deep anaesthesia, mice were perfused with 20 ml of 0.12 M cacodylate buffer followed by 30 ml of fixative made of 2.5% glutaraldehyde and 1% PFA (electron microscopy grade, EMS nos. 16221 and 15713) in cacodylate buffer (pH 7.4). Brains were removed and post-fixed for 1 h in the same fixative. Next, 300-μm-thick sagittal slices were made using a vibratome (VT 1000S, Leica Biosystems). Slices were post-fixed with 1% osmium tetroxide in 0.12 M cacodylate buffer for 2 h at room temperature and ‘en block’ stained with 1% uranyl acetate in 0.1 M acetate buffer overnight at room temperature. After several washes in acetate buffer, slices were dehydrated by passing them through increasing concentrations of ethyl alcohol (up to 100%). Slices were then progressively infiltrated in epoxy resin and placed for 48 h at 60 °C for resin polymerization. Then, 100-nm-thick sagittal ultrasections were performed using an ultramicrotome (Ultracut UC7, Leica Biosystems) through the whole cerebellum (+ brainstem) or the whole anterior brain (for corpus callosum analyses).

#### Image acquisition

Large ultrathin sections absorbed on silicon were observed with a FEI Helios NanoLab 660 FIBSEM field emission scanning electron microscope (FEI, Thermo Fisher Scientific) using high-resolution immersion mode and equipped with a solid-state concentric ring (insertable) back-scattering electron detector. Image acquisition was done using MAPS Software version 3.7 (Thermo Fisher Scientific). Low-magnification images (×600) were first taken to delineate our regions of interest (vermal inter-lobule 6–7 of the cerebellum). Then, high magnifications of two-dimensional image registration (×20,000–50,000) were taken using 4 kV/0.2 nAmp landing electron beam.

#### Myelin thickness measurement

The outer diameter (including myelin sheath) and inner diameter of at least 400 randomly selected myelinated axons were measured on one ultrathin section per animal (three animals per group). The g-ratio (equal to the ratio of the inner-to-outer diameter of a myelinated axon) of each axon was calculated. Scatter plots of the axon caliber and g-ratios were then analyzed. The slopes and elevations of the linear regressions from C and plKO mice were compared.

### Western blots

Whole mouse cerebellums and human vermal samples were homogenized in 250 μl of radioimmunoprecipitation assay lysis buffer consisting of (in mM) 50 Tris-HCl, pH 7.4, 150 NaCl, 2 EDTA, 50 NaF, 1 Na_3_VO_4_, 1% Triton X-100, 0.1% SDS, 0.5% Na-deoxycholate and a Protease/Phosphatase Inhibitor Cocktail (Santa Cruz Biotechnology). After centrifugation at 14,000*g* for 10 min, protein concentration was determined using Bradford protein assay kit (Bio-Rad). Sample total proteins were resolved by sodium dodecyl sulfate-polyacrylamide gel electrophoresis using 10% Bis-Tris precast gel (Thermo Fisher Scientific) and transferred to polyvinylidene fluoride membranes. Membranes were incubated with blocking buffer consisting of 4% non-fat milk in 1% Tween-20 in Tris-buffered saline (TBS-T) for 1 h, followed by overnight incubation at 4 °C with one of the following primary antibodies diluted in 3% BSA TBST-T: rabbit anti-MBP (Abcam, no. ab40390, 1:500), rabbit anti-MAG (Thermo Fisher Scientific, no. PA5-79620, 1:500), rabbit anti-MOG (Abcam, 1:500), mouse anti-cofilin-1 (Cfl1) (Santa Cruz Biotechnology, no. sc-53934, 1:500), mouse anti-prosaposin (Santa Cruz Biotechnology, no. sc-390184, 1:500), rabbit anti-hnRNPκ (R332) (Cell Signaling Technology, no. 4675, 1:500) and rabbit anti-GAPDH (Cell Signaling Technology, no. 5174, 1:2,000). After three washes with TBS-T, membranes were incubated with horseradish peroxide-conjugated secondary antibodies (Jackson ImmunoResearch, 1:2,000), and protein bands were visualized using the chemiluminescent ECL detection system (Bio-Rad) according to the manufacturer’s instruction. Signal intensities of protein bands were quantified using Image J software (http://rsb.info.nih.gov/ij/) and normalized with GAPDH as an internal control.

### Behavioral testing

Tests were performed on 30- and 60-d-old mice. No more than three behavioral tests were done on each mouse, accordingly to our Institutional Animal Care and Use Committee recommendations. A minimal resting period of 3 d was allowed between two consecutive tests.

#### Pup ultrasonic vocalizations

Mouse pups emit USVs when separated from the dam. This innate behavior is essential for eliciting maternal care behaviors that the pups need to survive. Separation-induced vocalizations were tested on postnatal days 4, 6, 8 and 11, with vocalizations declining drastically after P12. USVs were recorded for 3 min, and the number of calls was tracked using Avisoft-SASLab Pro software (Avisoft Bioacoustics).

#### Erasmus ladder

The Erasmus ladder (Noldus) is a fully automated system allowing for the assessment of baseline gait and locomotor coordination and associative cerebellar learning. The instrument consists of a horizontal ladder with 2 × 37 pressure-sensitive rungs for both the left and right sides, stationed between two shelter boxes. Each shelter is equipped with a white LED spotlight and pressurized air outlet serving as cues to signal time to departure from shelters. As the mouse is prompted to move, pressure sensors continuously monitor and analyze the walking pattern of the mouse in real time, sending data to the computer system, which, in turn, adjusts air pressure, calculates interventions and predicts future steps. Through this prediction, the software can also be programmed to move rungs mid-trial by high-speed pneumatic slides to create an obstacle. Mice were trained with 42 runs per day for four successive days without obstacles. Missteps, trial times and jumps were measured for each run. After training, cerebellar associative motor learning was assessed on days 4–8 with the introduction of a tone as a conditioning stimulus (CS) and a rising rung (obstacle) as the unconditioned stimulus (US). These perturbations were used to evaluate mouse ability to adapt motor behavior in real time. Repeated perturbations were used to assess associative motor learning throughout successive trials. With the inter-stimulus interval fixed at 285 ms, mice that associated the tone with the obstacle learned to increase walking speed to avoid being hit. In this sense, they decreased their ‘pre-perturbation step time’ (step before US) and their ‘post-perturbation step time’ (step just after the US). The Erasmus ladder is particularly valuable for detailed phenotyping of deficits associated with cerebellar pathology^17^.

#### Rotarod

The accelerating rotarod performance test is a standard rodent assay for motor-associated functions such as coordination and balance. A 4-d testing paradigm (three sessions per day) was performed as previously described^23^ with a continuously accelerating rod that forces animals to modulate their balance. For the first 2 d, animals were placed on the rod, which accelerated from 4 to 40 r.p.m. in 180 s. The test was stopped when the animal fell off the rod, was unable to maintain coordinated movements or 180 s passed. Additionally, if animals displayed ‘cartwheeling’, or clutching the rod and circling without walking, the test was stopped. To increase the challenge, for days 3–4 the acceleration of the rod increased from 8 to 80 r.p.m. in 180 s. The latency to fall from the rod and terminal velocity were then recorded. Linear regression analysis on data from each individual mouse was used to estimate initial motor coordination (given by the intercept) and learning rate (given by the slope).

#### Spontaneous behavior/stereotypies

Assessment of repetitive behavior was done to test autism-like behavior. Mice were allowed to habituate to the testing room before placement in a new, empty cage with bedding materials. A video camera was set with a lateral view to record spontaneous behavior for 15 min. Videos were manually scored for time spent engaging in behaviors, including digging, gnawing, grooming, rearing and jumping.

#### Marble burying

The anxiety and stereotypic and/or obsessive–compulsive-like behavior components of ASD-like behavior can be assessed in mice by their behavioral responses to new ‘diggable’ media^72,73^ in the marble burying test. This test was performed in a clean, large (26 ×16 cm) cage filled with 5 cm of bedding and 12 glass marbles evenly spaced on the bedding surface. The animal was left to explore the cage for 30 min undisturbed. Marbles covered 2/3 or more with bedding were counted as buried.

#### Socialization test

The three-chamber test (Crawley’s paradigm^74^) was used to assess social behavior as previously described^28^. A three-chamber Plexiglas box was used with small openings in each dividing wall to allow free access to each chamber. The center chamber was kept empty, wereas the flanking chambers were each equipped with an identical wire cup. After a 10-min habituation period to allow for exploration of the three-chamber equipment (session 1), an unfamiliar adult mouse of similar weight and coloration was placed within the wire cup in one of the side chambers and an ‘object’ within the wire cup in the opposite chamber. The dividers were raised to allow the test subject to move freely throughout all three chambers of the apparatus over a 10-min test session (session 2). The second session was analyzed for social preference given by the social preference index (SPI). SPI was calculated as follows: if time spent with the mouse (social) is S and the object (non-social) is NS, then SPI = S/(S + NS).

#### Determination of the autism composite severity score

We used the same procedure as previously described in ref. ^33^. The score calculation allows for the combination of individual discrete symptoms of the autistic syndrome, in a continuous manner, so that higher values reflect higher severity of autistic-like behavior. For each mouse, we recorded their spontaneous behaviors (grooming, digging, rearing, jumping and gnawing) and determined their SPI in the three-chamber test. The significant readouts (digging time, gnawing time and SPI) were then z-standardized and averaged to establish the autism composite severity score.

### Statistics and reproducibility

Mouse assignment to groups was not randomized, because it relies on their known genotype (C versus plKO), but mice from at least three different litters were used in each group for all experiments. No litter-associated effect was seen. All data analyses were conducted blinded to group allocation. Statistical analysis was carried out with GraphPad Prism 7 software. Detection of EYFP in Cyp19-cre:R26R-EYFP placenta and brain sections, and in situ hybridizations in placenta sections, were performed in at least three animals. Cell and area quantification on cerebellar immunofluorescent-labelled sections was done on six sections per animal, homogeneously distributed throughout the cerebellar vermis. Myelinated axon g-ratios were measured from more than 400 axons on one electron microscopy scan per animal. For western blot analyses, at least two technical replicates were performed. When more than ten samples, and, thus, several membranes, needed to be compared at once, two common samples were resolved in the different electrophoresis gels and used as reference for quantification. The sample sizes are shown in the legends and chosen to meet or exceed sample sizes typically used in the field. Robust regression and outlier removal methodology with Q value equal to 1 was used to determine outliers. Before conducting all analyses, variable distributions were evaluated for normality. If distribution was not normal, non-parametric methods were used. Differential gene expression across development in C and plKO mice was analyzed using one-way ANOVA with Dunnett’s multiple comparisons test. Comparisons between two groups were analyzed using parametric test (unpaired *t*-test with Welch’s correction) or non-parametric test (Mann–Whitney test). The Holm–Sidak method was used for multiple unpaired *t*-test comparison. Analyses involving data from three or more groups while considering only one independent variable were performed using one-way ANOVA with Tukey’s multiple comparisons test. Two-way ANOVA was used to compare means across two or more dependent variables. Two-way repeated-measures ANOVA with Sidak’s multiple comparisons tests were used for the Erasmus ladder and the rotarod. Summary data are presented in the text as mean ± s.e.m. from *n* animals. Differences were considered significant at *P* < 0.05. A supplementary statistics methods checklist is available.

### Reporting Summary

Further information on research design is available in the Nature Research Reporting Summary linked to this article.

## Online content

Any methods, additional references, Nature Research reporting summaries, source data, extended data, supplementary information, acknowledgements, peer review information; details of author contributions and competing interests; and statements of data and code availability are available at 10.1038/s41593-021-00896-4.

## Supplementary information

Reporting Summary

Supplementary Table 1 Steroid levels in the placenta at E12.5, E14.5 and E17.5. Two-way ANOVA with Sidak’s multiple comparisons test. The effect of *akr1c14* deletion in the placenta on ALLO levels was maximal at E14.5, when *akr1c14* gene expression peaks. No significant effect of sex was observed. ALLO, allopregnanolone; EPIALLO, epiallopregnanolone; PREG, pregnenolone; PROG, progesterone; ALLOTHDOC, allotetrahydrodeoxycorticosterone. E12.5: *n* = 5 independent samples per group. Comparison between male C and male plKO: ^α^*P* < 0.05, ^αα^*P* < 0.01, ^ααα^*P* < 0.005. Comparison between female C and female plKO: ^θ^*P* < 0.05, ^θθ^*P* < 0.01.

Supplementary Table 2 List of cerebellar DEGs in males (sheet 1) and females (sheet 2). Column A, gene Ensembl IDs; Column B, gene names; Column C, A values (average normalized counts); Column D, M values (normalized log fold changes); Column E, *P* values; Column F, *q* values; Column G, gene ranking.

Supplementary Table 3 Donors’ information for myelin/OL protein and gene expression analyses in infant vermis

## Data Availability

Raw and processed data are available from the corresponding authors upon reasonable request. The RNA-seq data discussed in this publication have been deposited in NCBI’s Gene Expression Omnibus (Vacher et al., 2021) and are accessible through GEO Series accession number GSE173440. Source data are provided with this paper.

## Source data

Source Data Fig. 2 Unprocessed western blots.
Source Data Fig. 5 Unprocessed western blots.
Source Data Extended Data Fig. 2 Unprocessed western blots.
