# Supplementary material for: Placental endocrine function shapes cerebellar development and social behavior
Source: Nat Neurosci. 2021 Aug 16;24(10):1392–401. doi: 10.1038/s41593-021-00896-4 (PMC8481124; doi:10.1038/s41593-021-00896-4)
Supplement: Supplementary file 1 — Reporting Summary [file 41593_2021_896_MOESM1_ESM.pdf]

nature research

Corresponding author(s): Anna Penn

Last updated by author(s): 05/28/2021

Reporting Summary

Nature Research wishes to improve the reproducibility of the work that we publish. This form provides structure for consistency and transparency in reporting. For further information on Nature Research policies, see our Editorial Policies and the Editorial Policy Checklist.

Statistics

For all statistical analyses, confirm that the following items are present in the figure legend, table legend, main text, or Methods section.

n/a	Confirmed	
☐	☒	The exact sample size (n) for each experimental group/condition, given as a discrete number and unit of measurement
☐	☒	A statement on whether measurements were taken from distinct samples or whether the same sample was measured repeatedly
☐	☒	The statistical test(s) used AND whether they are one- or two-sided<br>*Only common tests should be described solely by name; describe more complex techniques in the Methods section.*
☐	☒	A description of all covariates tested
☐	☒	A description of any assumptions or corrections, such as tests of normality and adjustment for multiple comparisons
☐	☒	A full description of the statistical parameters including central tendency (e.g. means) or other basic estimates (e.g. regression coefficient) AND variation (e.g. standard deviation) or associated estimates of uncertainty (e.g. confidence intervals)
☐	☒	For null hypothesis testing, the test statistic (e.g. F, t, r) with confidence intervals, effect sizes, degrees of freedom and P value noted<br>*Give P values as exact values whenever suitable.*
☒	☐	For Bayesian analysis, information on the choice of priors and Markov chain Monte Carlo settings
☒	☐	For hierarchical and complex designs, identification of the appropriate level for tests and full reporting of outcomes
☐	☒	Estimates of effect sizes (e.g. Cohen's d, Pearson's r), indicating how they were calculated

Our web collection on statistics for biologists contains articles on many of the points above.

Software and code

Policy information about availability of computer code

Data collection	Image acquisition on the scanning electron microscope was done using MAPS Software version 3.7 (ThermoFisher).<br>Image acquisition on the confocal microscope was done with LAS X Life Science Microscope software version 2.7 (Leica Microsystems).<br>Image acquisition on the scanning fluorescent microscope was done using cellSens version 2.3 (Olympus).<br>FSL software package 5.0.11 (FMRIB, Oxford, UK) was used to generate fractional anisotropy (FA) and mean diffusivity (MD) maps.
Data analysis	Data was analyzed using ImageJ 1.53c (NIH), Microsoft Excel 2016 and Graphpad Prism 7. Sequencing data was analyzed using TCC R package (version 1.12.1), Partek Genomics Suite (version 6.6), Ingenuity Pathway Analysis (IPA, Qiagen) and Venny 2.1.

For manuscripts utilizing custom algorithms or software that are central to the research but not yet described in published literature, software must be made available to editors and reviewers. We strongly encourage code deposition in a community repository (e.g. GitHub). See the Nature Research guidelines for submitting code & software for further information.

Data

Policy information about availability of data

All manuscripts must include a data availability statement. This statement should provide the following information, where applicable:

- Accession codes, unique identifiers, or web links for publicly available datasets
- A list of figures that have associated raw data
- A description of any restrictions on data availability

Raw and processed data are available from the corresponding authors upon request.

Field-specific reporting

Please select the one below that is the best fit for your research. If you are not sure, read the appropriate sections before making your selection.

☒ Life sciences ☐ Behavioural & social sciences ☐ Ecological, evolutionary & environmental sciences

For a reference copy of the document with all sections, see nature.com/documents/nr-reporting-summary-flat.pdf

Life sciences study design

All studies must disclose on these points even when the disclosure is negative.

Sample size

The sample sizes are shown in the legends and chosen to meet or exceed sample sizes typically used in the field (El Khordi et al., 2013; Lerch et al., 2011; Zonouzi et al., 2015). Preliminary experiments were performed when possible to determine requirements for sample size, taking into consideration resources available and ethical, reductionist animal use. Sample size was somewhat limited by the litter sizes and Mendelian ratios of complex genotypes but mice from at least 3 litters were used to overcome this limitation.

Data exclusions

- Effect of muscimol treatment on social behavior: one mouse was excluded because it refused to get out of the middle chamber in the 3-chamber equipment.
- Erasmus Ladder: 5 mice were removed from the experiment because they refused to perform the 42 runs in a row per day required for the 2-way Repeated Measures ANOVA analysis.
- Western blot on human tissue: One sample was excluded due to poor tissue quality.
- ROUT methodology was utilized to determine outliers.

Replication

Every experiment was performed on animals from at least 3 different litters. Two technical replicates were performed for RT-PCR and Western bot analyses on mouse and human samples. All attempts at replication were successful.

Randomization

Sample assignment to groups was not randomized, since it relies on their known genotype (C vs plKO mice) or gestational age (T and PT infants). Internal controls (housekeeping proteins and genes) were used in the molecular and biochemical experiments.

Blinding

All data collection and analyses were conducted blind to group allocation.

Reporting for specific materials, systems and methods

We require information from authors about some types of materials, experimental systems and methods used in many studies. Here, indicate whether each material, system or method listed is relevant to your study. If you are not sure if a list item applies to your research, read the appropriate section before selecting a response.

Materials & experimental systems

n/a	Involved in the study	
☐	☒	Antibodies
☒	☐	Eukaryotic cell lines
☒	☐	Palaeontology and archaeology
☐	☒	Animals and other organisms
☐	☒	Human research participants
☒	☐	Clinical data
☒	☐	Dual use research of concern

Methods

n/a	Involved in the study	
☒	☐	ChIP-seq
☒	☐	Flow cytometry
☐	☒	MRI-based neuroimaging

Antibodies

Antibodies used

Antibodies were used as follows for immunohistochemistry: : rabbit anti-MBP (Abcam, Cambridge, MA, USA; #ab40390; 1:500), mouse anti-APC clone CC1 (EMD Millipore, Burlington, NA, USA; #MABC20; 1:500), rabbit anti-Olig2 (Abcam; #ab9610, 1:500), mouse anti-neuronal nuclei (NeuN) (Millipore; #MAB377; 1:500), goat anti-NeuroD1 (RD Systems, Minneapolis, MN, USA; #AF2746; 1:500), rabbit anti-calbindin (Swant, Marly, Switzerland; #CB38, 1:1000), rat anti-mouse PDGFRα (CD140a; BD Biosciences, San Jose, CA, USA; #17-1401-81; 1:500), chicken anti-GFP (Abcam; #ab13970; 1:500), , donkey anti-mouse Alexa-488 (Invitrogen; #A-21202), donkey anti-rabbit Alexa-488 (Invitrogen; #A-21206), donkey anti-mouse Alexa-555 (Invitrogen; #A-31570), donkey anti-rabbit Alexa-555 (Invitrogen; #A-31572), donkey anti-mouse Alexa-647 (Invitrogen; #A-31571), and donkey anti-rabbit Alexa-647 (Invitrogen; #A-31573).
Antibodies were used as follows for Western blots: rabbit anti-MBP (Abcam; #ab40390; 1:500), rabbit anti-MAG (Thermo Fisher Scientific; #PA5-79620; 1:500), rabbit anti-MOG (Abcam; #ab32760; 1:500), mouse anti-cofilin-1 (Cfl1) (Santa Cruz Biotechnology, Dallas, TX, USA; #sc-53934; 1:500), mouse anti-prosaposin (PSAP) (Santa Cruz Biotechnology; #sc-390184; 1:500), rabbit anti-hnRNPκ (R332) (Cell Signaling Technology; #4675; 1:500) and rabbit anti-GAPDH (Cell Signaling Technology, Danvers, MA, USA; #5174; 1:2000).

Validation

These antibodies were commercially available and validated as noted on the manufacturers website. Additional verifications were

Validation | done as follows. The specificity of the secondary fluorescent antibodies was checked by omitting the primary antibodies. The validity of the primary antibodies was tested with the molecular weight of the bands detected by Western blot in mouse and human samples. The antibodies (or clones) are referenced in the Antibodyregistry.org.

Animals and other organisms

Policy information about studies involving animals; ARRIVE guidelines recommended for reporting animal research

Laboratory animals	The strains used in this study were: Cyp-19a-Cre, Akr1c14-floxed and ROSA26-YFP mice. Males and females were collected at embryonic days: E12.5, E14.5, E15.5, E16.5, E17.5, E19.5 and postnatal days P30.<br>Mice were housed in a climate controlled facility with a fixed 12/12 hour light cycle with ad libitum access to food and water. All behavioral experiments were conducted between 8 am and 5 pm.
Wild animals	The study did not involve wild animals.
Field-collected samples	The study did not involve samples collected from fields.
Ethics oversight	All the procedures on experimental animals were performed in accordance with the protocols approved by the Institutional Animal Care and Use Committee at Children's National Medical Center (protocol # 30534 (PI: Penn)) and at Columbia University Medical Center (protocols # AC-AABE 6553 (PI: Penn) and AC-AABF 5550 (PI: Yang)).

Note that full information on the approval of the study protocol must also be provided in the manuscript.

Human research participants

Policy information about studies involving human research participants

Population characteristics	*Describe the covariate-relevant population characteristics of the human research participants (e.g. age, gender, genotypic information, past and current diagnosis and treatment categories). If you filled out the behavioural & social sciences study design questions and have nothing to add here, write "See above."*
Recruitment	*Describe how participants were recruited. Outline any potential self-selection bias or other biases that may be present and how these are likely to impact results.*
Ethics oversight	*Identify the organization(s) that approved the study protocol.*

Note that full information on the approval of the study protocol must also be provided in the manuscript.

Magnetic resonance imaging

Experimental design

Design type	MRI was used to examine the structural integrity of white matter tracts in the brains of 4% paraformaldehyde fixed brains.
Design specifications	4% paraformaldehyde perfused brains
Behavioral performance measures	no tasks performed during

Acquisition

Imaging type(s)	structural/diffusion
Field strength	7T
Sequence & imaging parameters	Parameters for the DTI sequence were as follows: TR of 270 ms, echo train length of 6, first TE of 32 ms, and a TE of 10 ms for the remaining 5 echoes, 1 average, FOV of 14 mm x 14 mm x 25 mm and a matrix size of 180 x 180 x 324 resulting in an image with 78 µm isotropic voxels. Five b=0 s/mm2 images and 30 high b value (b=2147 s/mm2) in 30 different directions were acquired, using the Jones scheme. Total imaging time was approximately 12 hours.
Area of acquisition	whole brain

Diffusion MRI ☒ Used ☐ Not used

Parameters | Parameters for the DTI sequence were as follows: TR of 270 ms, echo train length of 6, first TE of 32 ms, and a TE of 10 ms for the remaining 5 echoes, 1 average, FOV of 14 mm x 14 mm x 25 mm and a matrix size of 180 x 180 x 324 resulting in an image with 78 µm isotropic voxels. Five b=0 s/mm2 images and 30 high b value (b=2147 s/mm2) in 30 different directions were acquired, using the Jones scheme. Total imaging time was approximately 12 hours.

Preprocessing

Preprocessing software	FSL software package (FMRIB, Oxford, UK)

Normalization	To assess any changes to the mouse brains due to genotype and sex, the b=0 s/mm2 images were registered linearly (6 followed by 12 parameter) and non linearly together. A combination of mni_autoreg tools and ANTS (advanced normalization tools) were used to perform the registrations.
Normalization template	A population atlas, representing the average anatomy of the study samples, was created when all scans were resampled with an appropriate transform. The final registration results are the individual images deformed into alignment with one another in an unbiased manner. Warping an available classified MRI mouse brain atlas onto the population average allows for significant differences in the diffusion measures of segmented structures to be calculated.
Noise and artifact removal	N/A
Volume censoring	N/A

Statistical modeling & inference

Model type and settings	This study examined fixed brains, without any behavioral tasks, no statistical modeling was involved.
Effect(s) tested	The analysis of the individual DTI parameters compare intensity differences of FA between genotype and sex, in different ROIs, using 3-way ANOVA.

Specify type of analysis: ☐ Whole brain ☒ ROI-based ☐ Both

Anatomical location(s)	- Cerebellum-related tracts<br>- Other major cerebral tracts: anterior commissure, posterior commissure, corpus callosum, internal capsule, fornix, mammillothalamic tract, corticospinal tract, cerebral peduncle.
Statistic type for inference (See Eklund et al. 2016)	Statistical analysis was based on brain region clusters: cerebellar WM tracts and other major (extra-cerebellar) WM tracts
Correction	N/A

Models & analysis

n/a	Involved in the study
☒	☐ Functional and/or effective connectivity
☒	☐ Graph analysis
☒	☐ Multivariate modeling or predictive analysis
