# Supplementary material for: Placental endocrine function shapes cerebellar development and social behavior
Source: Nat Neurosci. 2021 Aug 16;24(10):1392–401. doi: 10.1038/s41593-021-00896-4 (PMC8481124; doi:10.1038/s41593-021-00896-4)

Source Data Fig. 2a

Males

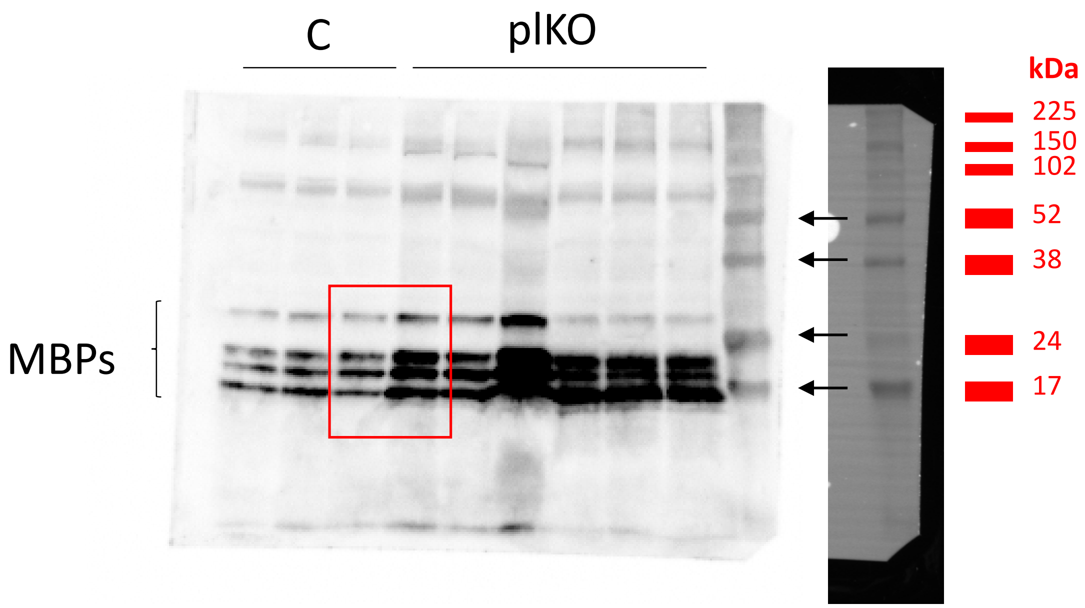

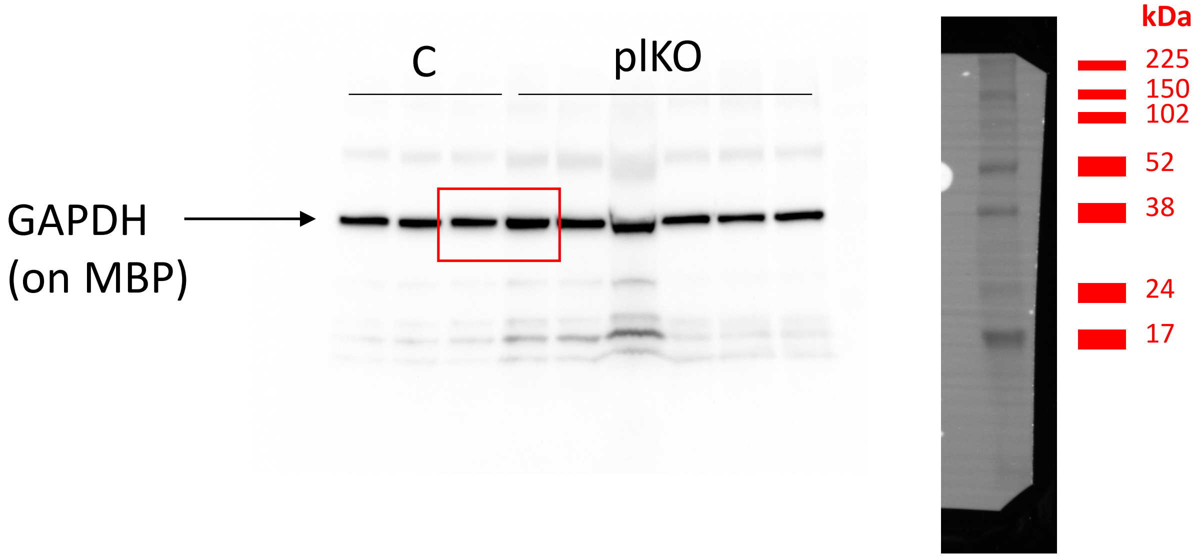

Source Data Fig. 2a

Males

Sample order

1 2 3 4 5 6 7 8 9

C – C - plKO – plKO – C – plKO – C – plKO – plKO

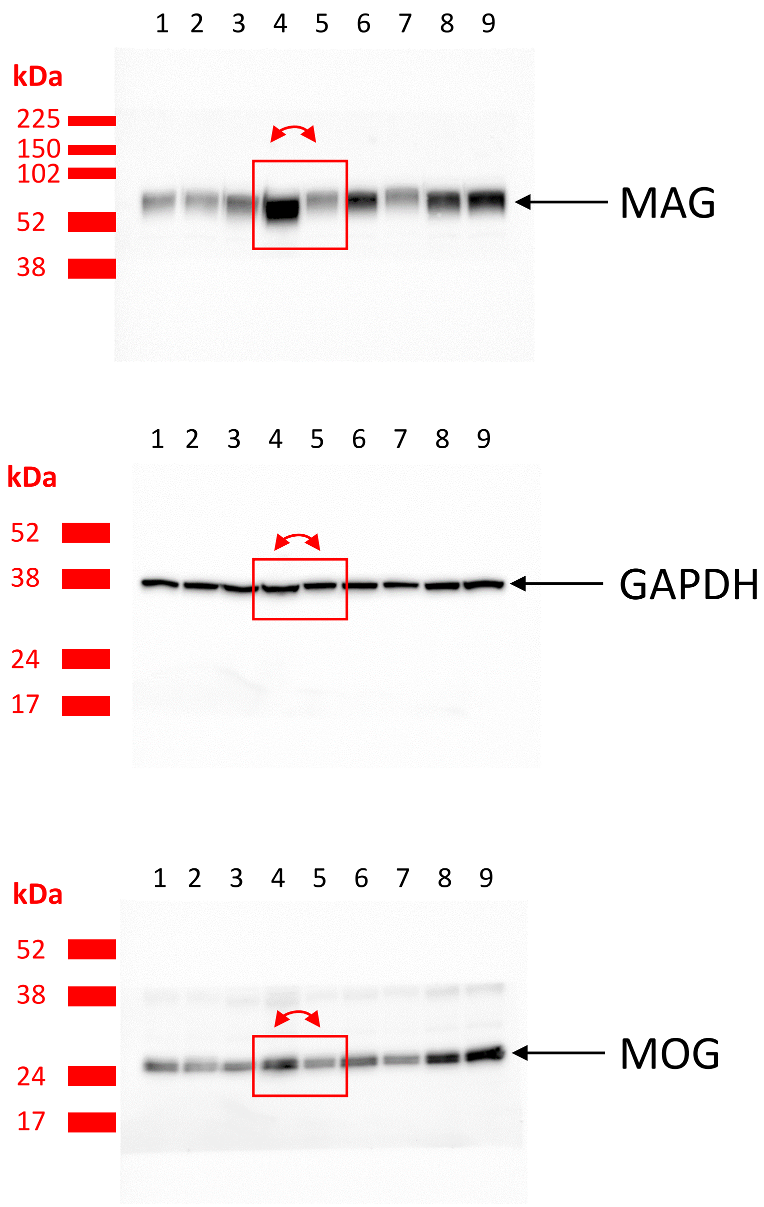

Source Data Fig. 2c

Females

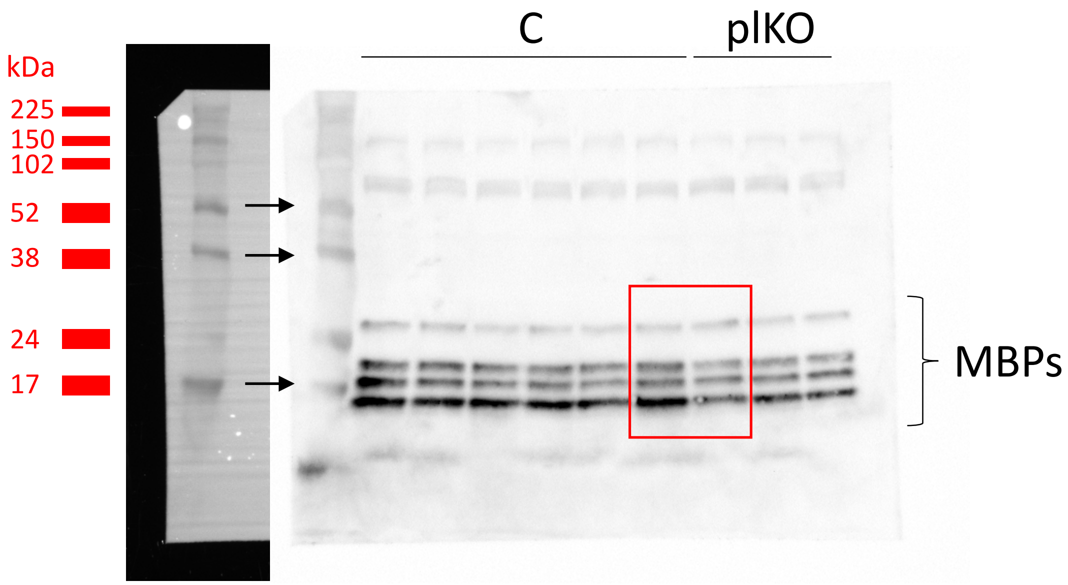

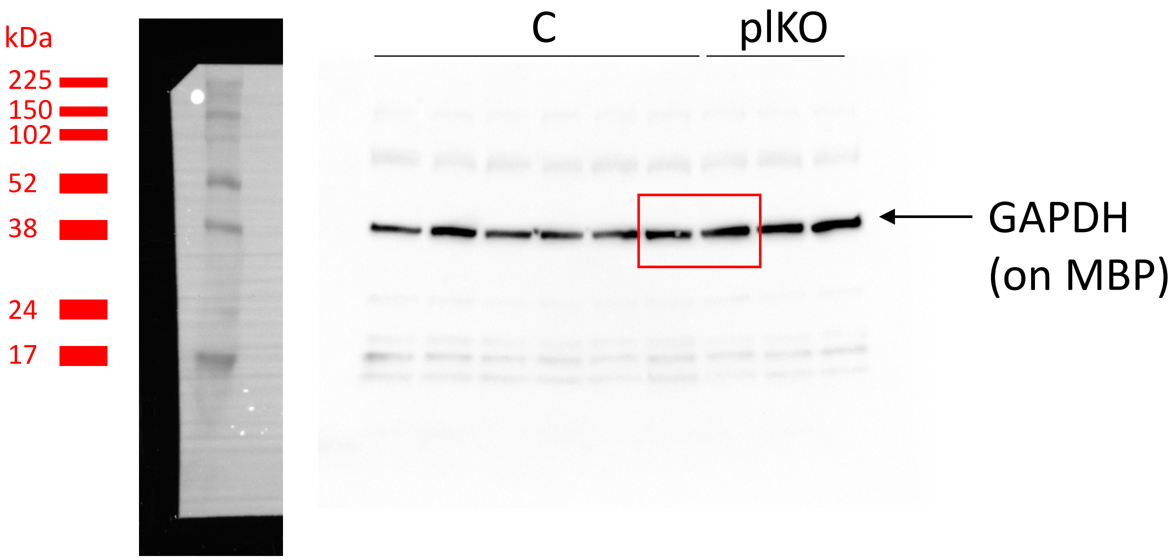

Source Data Fig. 2c

Females

Sample order

1 2 3 4 5 6 7 8 9

C – plKO - C – plKO – C – plKO – C – plKO – C

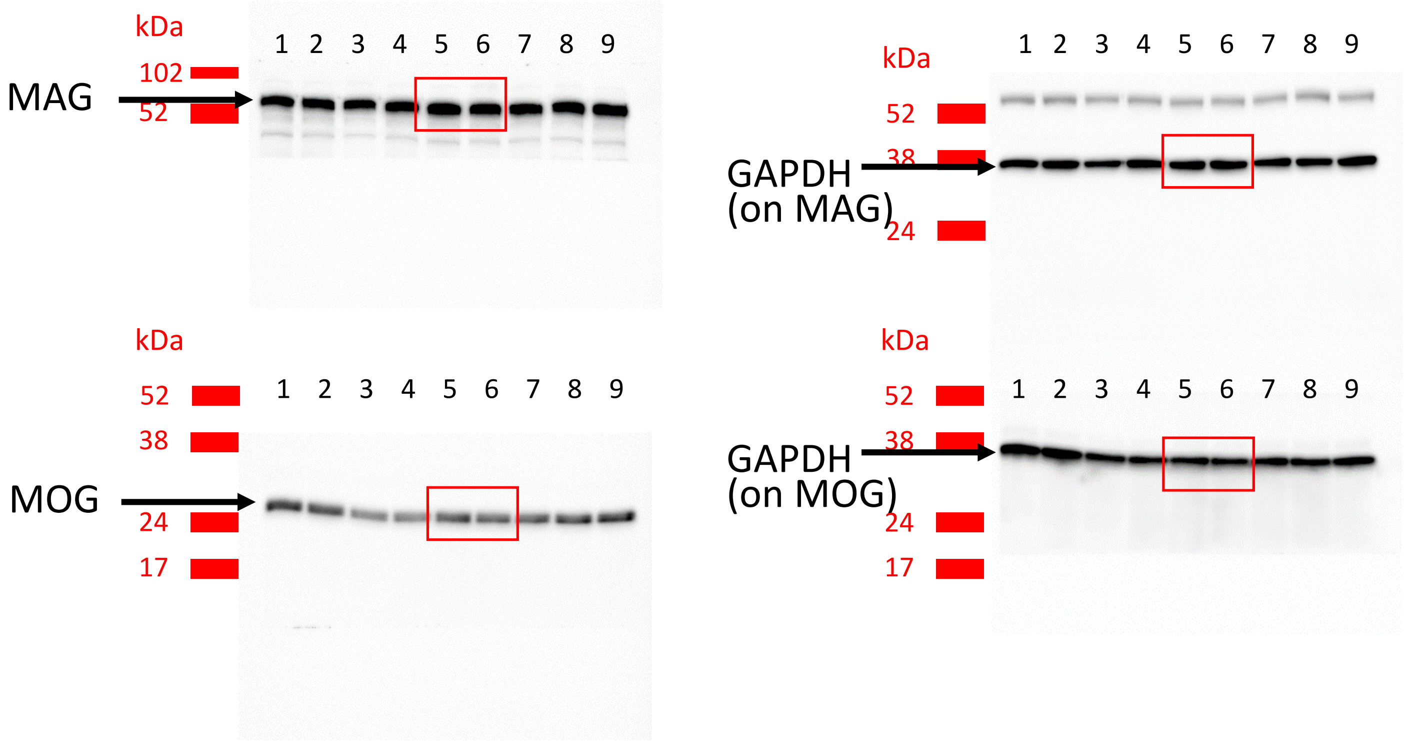

Supplement: Source Data Fig. 2 — Unprocessed western blots. [file 41593_2021_896_MOESM5_ESM.pdf]
