# Supplementary material for: Placental endocrine function shapes cerebellar development and social behavior
Source: Nat Neurosci. 2021 Aug 16;24(10):1392–401. doi: 10.1038/s41593-021-00896-4 (PMC8481124; doi:10.1038/s41593-021-00896-4)

Source Data Fig. 5

Females

Sample order

1 2 3 4 5 6 7 8 9 10
T - PT - T – PT – T – PT – T – PT – PT – PT

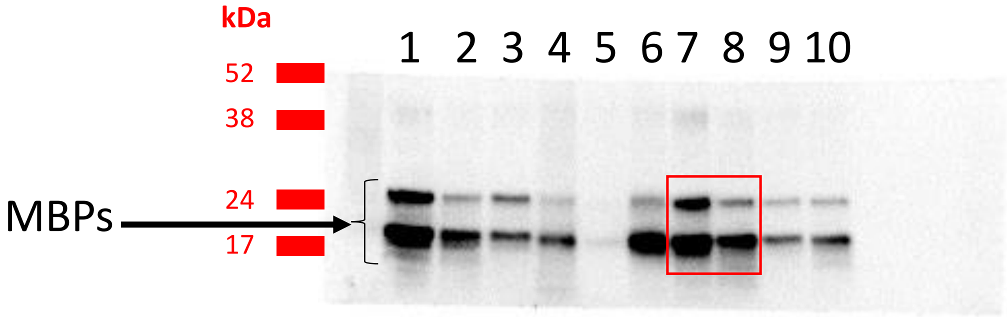

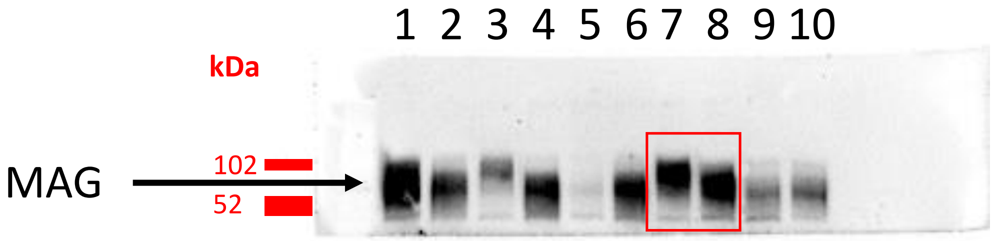

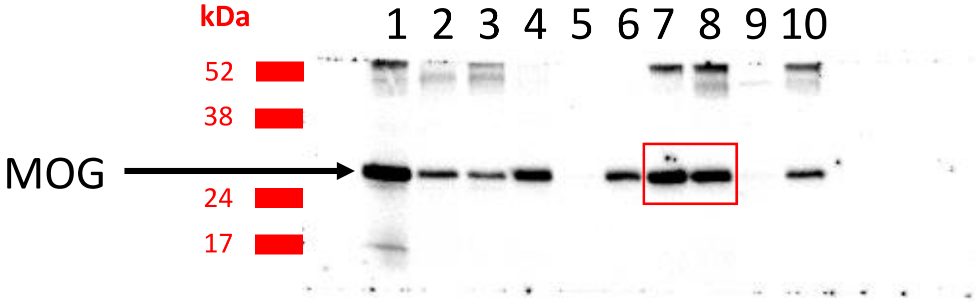

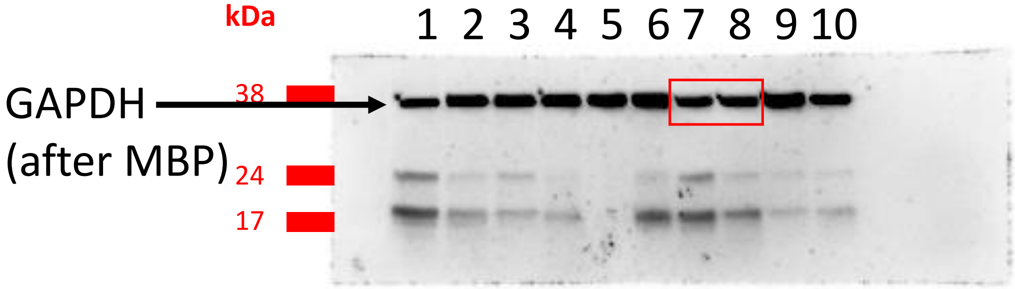

Source Data Fig. 5

Males

Sample order

1 2 3 4 5 6 7 8 9 10 11

T - PT - T – PT – T – PT – PT – T – PT – T - T

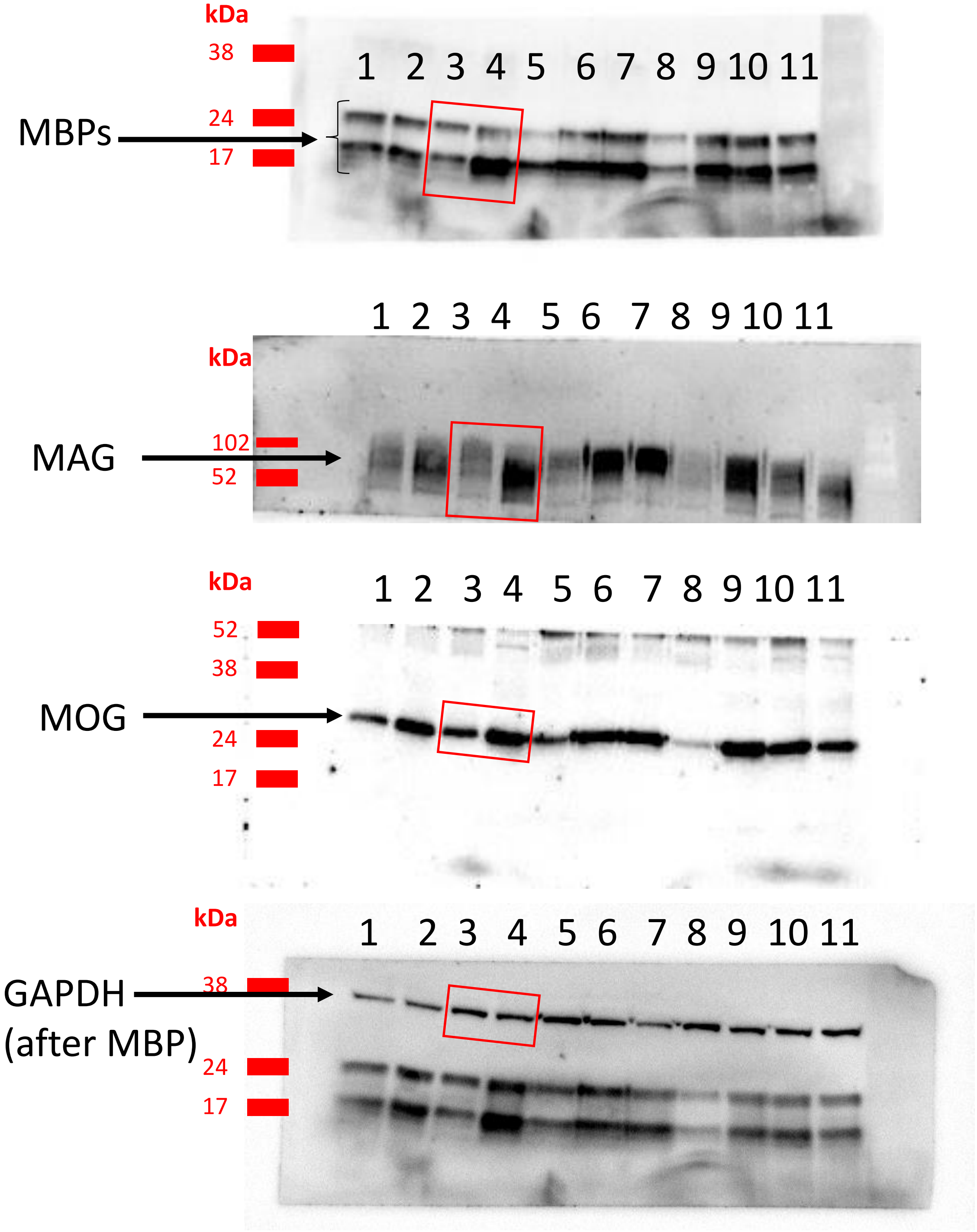

Supplement: Source Data Fig. 5 — Unprocessed western blots. [file 41593_2021_896_MOESM6_ESM.pdf]
