# Supplementary material for: Placental endocrine function shapes cerebellar development and social behavior
Source: Nat Neurosci. 2021 Aug 16;24(10):1392–401. doi: 10.1038/s41593-021-00896-4 (PMC8481124; doi:10.1038/s41593-021-00896-4)

Source Data Extended Data Fig. 2

Sample order

1 2 3 4 5 6 7 8

C – plKO- C – plKO– C – plKO – C – plKO

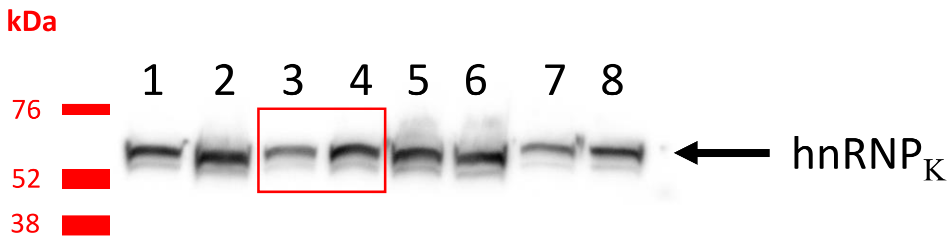

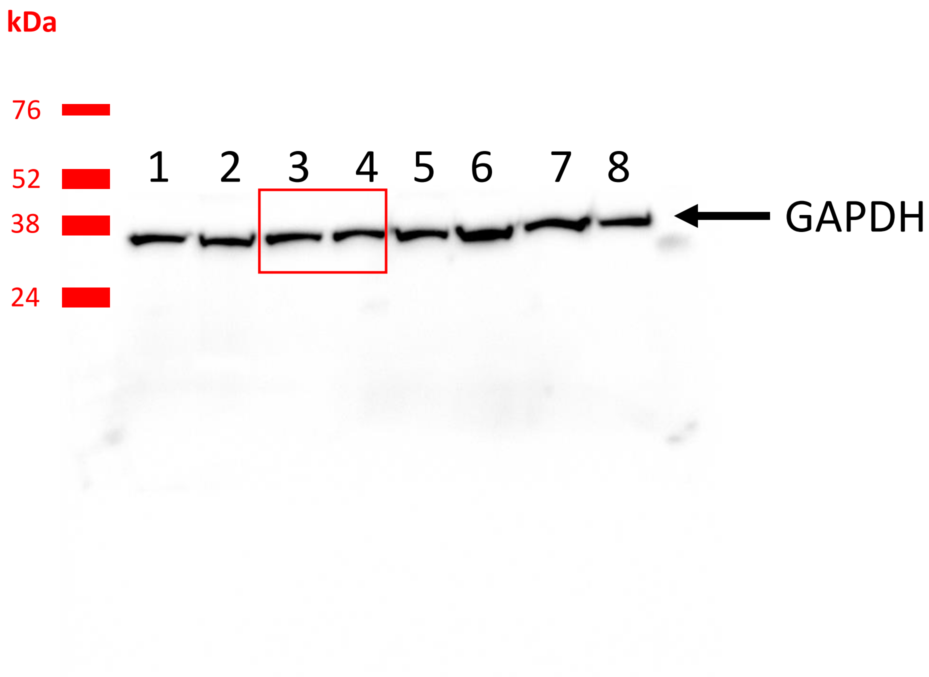

Source Data Extended Data Fig. 2

Sample order

1 2 3 4 5 6 7 8
C – C- plKO – plKO– C – C – plKO - C

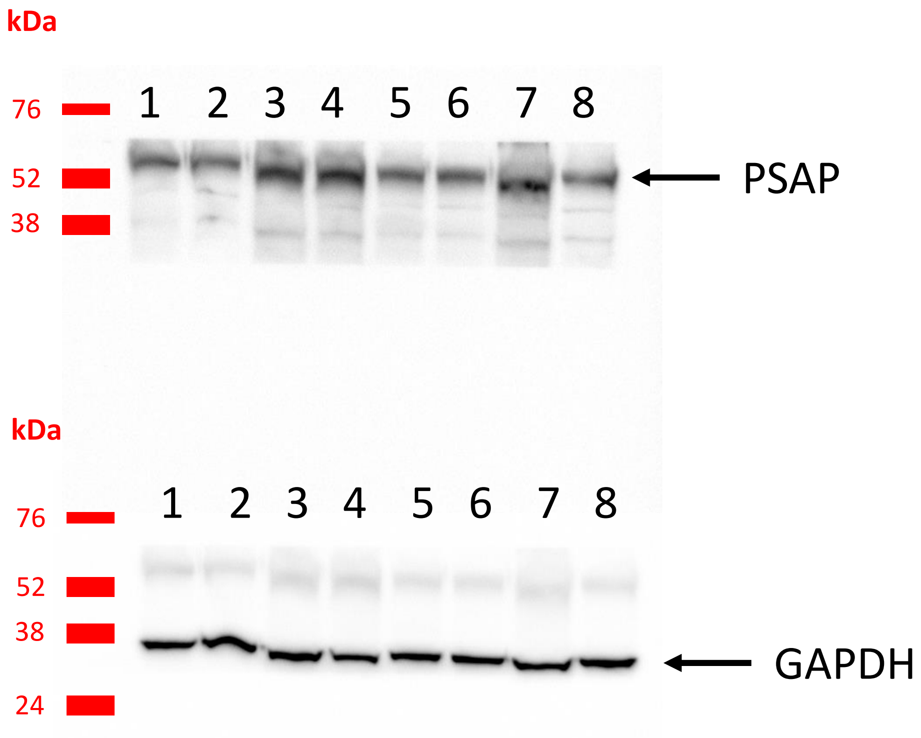

Source Data Extended Data Fig. 2

Sample order

1 2 3 4 5 6 7 8

C – plKO - C – plKO – C – plKO – C - plKO

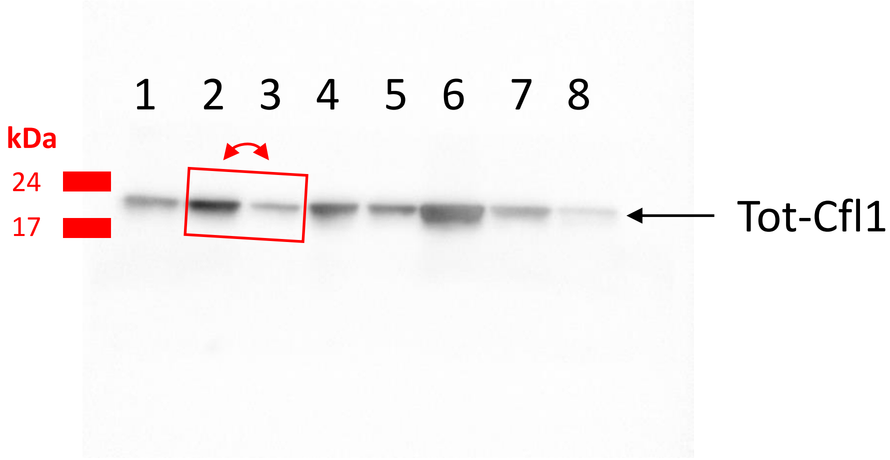

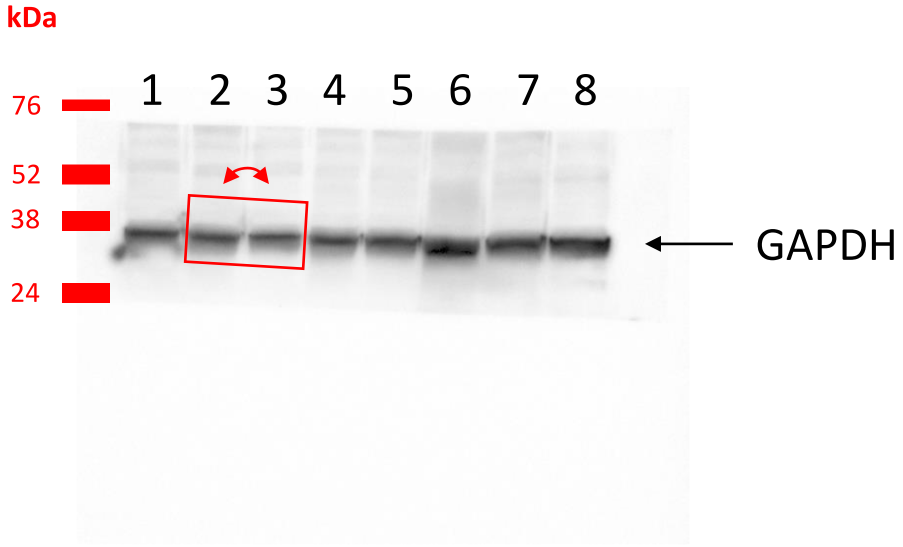

Supplement: Source Data Extended Data Fig. 2 — Unprocessed western blots. [file 41593_2021_896_MOESM7_ESM.pdf]
